# Antifungal metabolites from *Myxococcus stipitatus* GXUA 01510 for the control of sugarcane Pokkah boeng disease caused by *Fusarium sacchari*

**DOI:** 10.1039/d5ra10061e

**Published:** 2026-05-06

**Authors:** Wang Jiang, Qun Hao Li, Jiasong Guan, Ahmed F. Elkarmout, Zhonghui Ma, Zhiwei Su

**Affiliations:** a National Demonstration Center for Experimental Plant Science Education, College of Agriculture, Guangxi University Nanning 530004 Guangxi China mazhonghui@gxu.edu.cn suzhiwei@gxu.edu.cn; b Department of Horticulture, Faculty of Agriculture, Tanta University Tanta Egypt

## Abstract

Four previously undescribed phenyl polyenes, prophenalamides D-E (1–2) and phenalamides F-G (3–4), along with nine known compounds (5–13), were isolated from the fermentation extract of *Myxococcus stipitatus* GXUA 01510. Compounds 10–13 have not previously been reported from myxobacteria. The structures of these compounds were elucidated using HR-ESI-MS, and NMR spectroscopic analyses. The antifungal experiment revealed that the thiazole compounds cystothiazole A (7) and melithiazol F (9) exhibited significant activity against *Fusarium sacchari* strains FS-9DF-3-2 and FS-2-1, an agricultural pathogen associated with sugarcane Pokkah boeng disease. Cystothiazole A (7) significantly inhibited spore germination, displaying EC_50_ values of 1.00 µg mL^−1^ against FS-9DF-3-2 and 0.67 µg mL^−1^ against FS-2-1. Regarding mycelial growth, it exhibited EC_50_ values of 3.01 and 9.71 µg mL^−1^ for the two strains. Remarkably, unlike the positive control and many other antifungal natural products, cystothiazole A (7), which is a rare trait among existing agents, exhibits a distinctive ability to suppress spore germination. Similarly, melithiazol F (9) inhibited mycelial growth, with EC_50_ values of 2.94 and 1.08 µg mL^−1^ against FS-9DF-3-2 and FS-2-1, respectively. Furthermore, both cystothiazole A (7) and melithiazol F (9) significantly increased nucleic acid and protein leakage in *F. sacchari* spores, highlighting their potential to disrupt cell membrane integrity. These findings not only enrich the chemical diversity of myxobacterial metabolites but also provide promising molecular scaffolds for developing novel biocontrol agents.

## Abstract 2

The thiazole compounds cystothiazole A (7) and melithiazol F (9) potently inhibited *Fusarium sacchari* by suppressing its growth and spore germination and disrupting cell membrane integrity.

## Graphical abstract

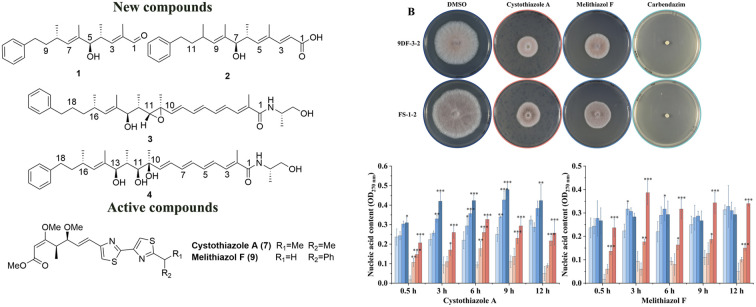

## 1 Introduction

Myxobacteria are Gram-negative, rod-shaped bacteria widely recognized for their social behavior.^1^ They exhibit complex multicellular behaviors, such as gliding motility, group predation, and the formation of distinct fruiting bodies, and are considered advanced prokaryotes and essential model organisms for scientific research.^2–4^ Furthermore, myxobacteria are emerging as a prolific source of bioactive compounds, with over 816 secondary metabolites identified to date.^5^ Several of these compounds exhibit potent inhibitory activity against plant pathogenic fungi, positioning them as promising candidates for the development agricultural antibiotic.^6–8^

Recent research into the biocontrol of myxobacteria has preliminarily elucidated their antagonistic mechanisms, which primarily involve the secretion of lytic enzymes and antimicrobial secondary metabolites.^9^ For example, Dong *et al.*^10^ reported that *Myxococcus xanthus* R31 exhibits predatory activity against the tomato wilt pathogen *Ralstonia solanacearum* in plate assays, achieving 81.9% control efficacy against tomato bacterial wilt in pot experiments. Proteomic analysis identified nine lytic enzymes among the extracellular proteins of R31, underscoring the critical role of secreted proteins in its predatory behavior.^10^ Beyond proteins, secondary metabolites also play a pivotal role. In the study by Meliah *et al.*,^11^ ethyl acetate extracts from *Corallococcus* strains KR39b.5 and SLU3.3 inhibited the growth of *Fusarium odoratissimum*, the causal agent of Panama disease in bananas, by more than 40%. Similarly, Wu *et al.*^12^ demonstrated that active extracts from *Sorangium cellulosum* B25-I-1 significantly reduced the soluble protein content and antioxidant enzyme activity in *Phytophthora infestans*. These extracts were found to induce oxidative stress and increase membrane permeability, effectively inhibiting pathogen infection in detached potato leaves. Further analysis identified methyl (2*R*)-2-azido-3-hydroxy-2-methylpropanoate and *N*-(3-amino-2-hydroxypropyl)-*N*-methylsulfuric diamide as the major antagonistic components.^12^ Collectively, these findings highlight the considerable biocontrol potential of myxobacteria.

Sugarcane is a vital crop for sugar and energy production, accounting for approximately 70% of global sugar production and offering substantial potential for biomass and ethanol-based biofuel production.^13^ However, large-scale continuous cultivation has led to a surge in crop diseases, particularly Pokkah boeng disease mainly caused by *Fusarium sacchari*, which severely affects both yield and quality of sugarcane.^14^ The spread of sugarcane Pokkah boeng disease relies heavily on the dispersal of fungal spores.^15^ However, most prior studies have focused primarily on inhibiting the mycelial growth of the pathogen, with relatively few studies have examined spore germination. Among these, the tested fungicides have generally shown limited effectiveness.^16,17^ For example, Hu *et al.*^16^ reported that the antimicrobial peptide fengycins effectively inhibited *F. moniliforme* mycelial growth but delayed only spore germination without causing inactivation. In our prior studies, we reported that fermentation extracts from various myxobacterial strains strongly inhibited *F. sacchari*.^18^ Among them, *Myxococcus stipitatus* GXUA 01510 exhibited particularly promising antifungal activity. However, the chemical basis underlying its activity remains unclear, and the inhibitory mode of action of its active metabolites against *F. sacchari* has not been explored. Building on this, this study describes the isolation and identification of four undescribed phenyl polyenes and nine known compounds from the metabolites of *M. stipitatus* GXUA 01510. Among them, cystothiazole A (7) and melithiazol F (9) exhibited significant antifungal activity against *F. sacchari* strains. Furthermore, a preliminary investigation revealed that some of the isolates inhibited spore germination and mycelial growth of *F. sacchari*, accompanied by significant leakage of nucleic acids and proteins. By identifying the bioactive metabolites of *M. stipitatus* GXUA 01510 and preliminarily revealing their antifungal mode of action against *F. sacchari*, this study provides a clearer basis for exploiting myxobacterial metabolites in the biological control of sugarcane Pokkah boeng disease.

## 2 Results and discussion

### 2.1 Structural identification of new compounds

**Fig. 1:**
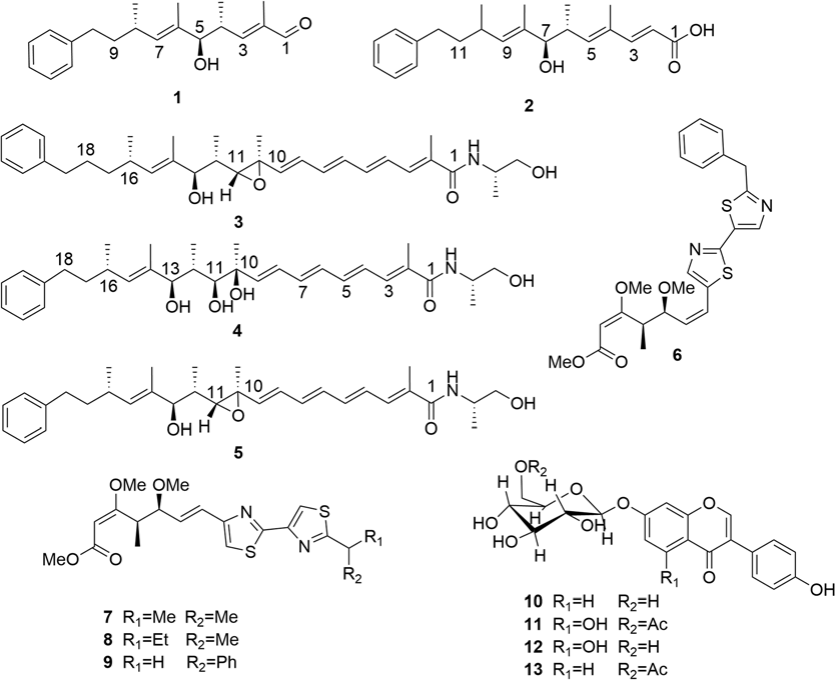
Structures of compounds 1–13.

Large-scale fermentation of *Myxococcus stipitatus* GXUA 01510 yielded 20 g of crude extract, which was subsequently subjected to separation and structural identification. This process led to the isolation of thirteen compounds, including four undescribed phenyl polyenes, prophenalamides D-E (1–2) and phenalamides F-G (3–4), along with nine known compounds (5–13), phenalamid C (5),^19^, (6*Z*)-melithiazol F (6),^20^ cystothiazole A (7),^21^ melithiazole G (8),^22^ melithiazol F (9),^23^ daidzin (10),^24^ acetyl-genistin (11),^25^ genistin (12),^26^ and acetyl-daidzin (13)^25^ (Fig. 1). Among them, the known compounds 10–13 were isolated from myxobacteria for the first time.

**Fig. 2:**
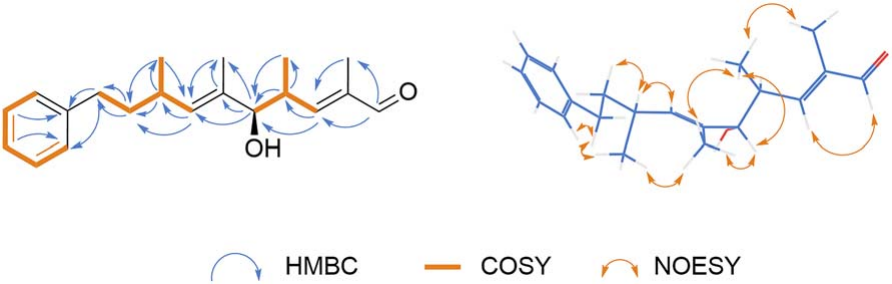
The key HMBC, COSY, and NOESY correlations of compounds 1.

Compound 1, obtained as a colorless resinous solid, was determined to have the molecular formula C_20_H_28_O_2_, as established by the HR-ESI-MS ion at *m*/*z* 323.1987 [M + Na]^+^ (*calcd*. 323.1983) and supported by the 1D and 2D NMR data. The IR spectrum exhibited characteristic absorptions for an *α*,*β*-unsaturated aldehyde (1675 cm^−1^), a benzene ring (1453, 747 and 699 cm^−1^).^27^ The ^1^H NMR spectrum (Table 1) revealed signals corresponding to an aldehyde proton [*δ*_H_ 9.43 (1H, s, H-1)], five benzene ring protons [*δ*_H_ 7.16–7.26 (5H, m, H-12 to H-16)], two olefinic protons [*δ*_H_ 6.46 (1H, dd, *J* = 9.6, 1.4 Hz, H-3), 5.24 (1H, d, *J* = 9.6 Hz, H-7)], four methyl signals [*δ*_H_ 1.79 (3H, d, *J* = 1.4 Hz, 2-Me), 0.95 (3H, d, *J* = 6.8 Hz, 4-Me), 1.60 (3H, d, *J* = 1.4 Hz, 6-Me), 0.98 (3H, d, *J* = 6.8 Hz, 8-Me)], two methylene signals [*δ*_H_ 1.53–1.70 (2H, m, H-9), 2.52–2.62 (2H, m, H-10)], and three methine signals [*δ*_H_ 2.90 (1H, m, H-4), 3.86 (1H, d, *J* = 8.0 Hz, H-5), 2.38–2.47 (1H, m, H-8)]. The ^13^C NMR spectrum displayed 20 carbon signals (Table 1), including one aldehyde carbon at *δ*_C_ 195.4 (C-1), six benzene carbons [*δ*_C_ 142.5 (C-11), 128.4 (C-12), 128.3 (C-13), 125.7 (C-14), 128.3 (C-15), 128.4 (C-16)], four olefinic carbons [*δ*_C_ 139.7 (C-2), 157.7 (C-3), 133.9 (C-6), 135.4 (C-7)], four methyl carbons [*δ*_C_ 9.6 (2-Me), 16.6 (4-Me), 11.5 (6-Me), 21.0 (8-Me)], two methylene carbons [*δ*_C_ 38.9 (C-9), 33.9 (C-10)] and three methine carbons [*δ*_C_ 37.2 (C-4), 81.9 (C-5), 31.7 (C-8)]. The sequential ^1^H–^1^H COSY correlations observed from H-3 to H-10 (Fig. 2), together with the ^1^H and ^13^C NMR data, indicate that the compound possesses a core structure featuring a monosubstituted benzene ring conjugated with an unsaturated branched polyene chain. Analysis of the HMBC spectrum reveals that the correlation between [*δ*_H_ 9.43 (1H, s, H-1)] and [*δ*_C_ 139.70 (C-2)] (Fig. 2), suggesting that this compound is a terminal aldehyde. The HMBC correlation (Fig. 2) between [*δ*_H_ 7.16 (3H, m, H-12, 14, 16)] and [*δ*_C_ 33.91 (C-10)] indicated a phenyl substitution at C-10. The characteristic olefinic proton signals [*δ*_H_ 6.46 (1H, dd, *J* = 9.6, 1.4 Hz, H-3), 5.24 (1H, d, *J* = 9.6 Hz, H-7)] were assigned to Δ^2^ and Δ^6^ double bonds, respectively. The simultaneous NOESY correlations (Fig. 2) were observed between H-1 and H-3, 2-Me and 4-Me, 6-Me and H-5, as well as H-7 and H-8, collectively suggested that the double bonds at C-2 and C-6 adopt the *E* configurations, thereby supporting the assignment of a 2*E*,6*E*-dienal moiety.

**Table 1:** ^1^H NMR and ^13^C NMR data of compounds 1 and 2 (in CDCl_3_)^a^

Position	1		2		
	*δ*_C_	*δ*_H_ (*J* in Hz)	Position	*δ*_C_	*δ*_H_ (*J* in Hz)
1	195.4	9.43, s	1	171.6	—
2	139.7	—	2	115.2	5.80, d (15.6)
3	157.1	6.46, dd (9.6, 1.4)	3	151.7	7.42, d (15.6)
*	—	—	4	133.8	—
*	—	—	5	145.7	5.87, d (9.8)
4	37.2	2.85–2.94, m	6	37.2	2.38–2.47, m
5	81.9	3.86, d (8.0)	7	82.2	3.77, d (8.1)
6	133.9	—	8	134.0	—
7	135.4	5.24, d (9.6)	9	135.2	5.22, d (9.5)
8	31.7	2.38–2.47, m	10	31.9	2.72–2.80, m
9	38.9	1.53–1.70, m	11	39.0	1.53–1.70, m
10	33.9	2.52–2.62, m	12	33.9	2.51–2.62, m
11	142.5	—	13	142.6	—
12	128.4	7.12–7.19, m	14	128.4	7.10–7.16, m
13	128.3	7.23–7.29, m	15	128.2	7.23–7.28, m
14	125.7	7.12–7.19, m	16	125.6	7.10–7.16, m
15	128.3	7.23–7.29, m	17	128.2	7.23–7.28, m
16	128.4	7.12–7.19, m	18	128.4	7.10–7.16, m
2-Me	9.6	1.79, d (1.4)	4-Me	16.6	1.85, d (1.4)
4-Me	16.6	0.95, d (6.8)	6-Me	17.1	0.89, d (6.8)
6-Me	11.5	1.60, d (1.4)	8-Me	11.6	1.60, d (1.4)
8-Me	21.0	0.98, d (6.8)	10-Me	21.0	0.97, d (6.8)

a ^1^H NMR spectra measured at 500 MHz, ^13^C NMR spectra measured at 125 MHz. ‘*’ represents that the space left out corresponds to other similar signals of another compound.

Recent studies employing the chemical space mapping (CSM) strategy emphasized that theoretical chemical space is strictly constrained by biosynthetic rules.^28^ Consistent with this, myxobacteria are known to possess highly conserved biosynthetic pathways.^5^ Therefore, based on this conservation and the structural similarity between compounds 1–4 and previously reported analogs, it is inferred that these molecules should have a consistent stereochemical configuration.^29^ Specifically, they likely arise from the oxidative degradation of aliphatic amino acids such as alanine, *α*-aminobutyric acid, valine, and isoleucine.^19^ Previous studies have proposed that, for phenalamides, the biosynthetic side chain is extended outward from the phenyl ring terminus (Fig. S1).^29^ In light of this, compound 1 is notably postulated to act as a biogenetic precursor of compound 2–4, and other related phenalamides (Fig. S1).^29^ We speculate that these compounds likely share the same configuration as the known compound phenalamide A_2_,^19^ namely 4*R*, 5*R*, and 8*S*. This hypothesis was corroborated by NOESY data (Fig. 2). Key correlations, specifically between 2-Me/4-Me, 4-Me/6-Me/H-5, 6-Me/8-Me/H-5, and H-8/H-7/H-9, indicated that the methyl groups at C-2, C-4, and C-6 are in close spatial proximity. The structure of phenalamide A_2_ has been well elucidated.^19^ Based on the high degree of consistency in the NMR data (Table S1) for the corresponding structural fragments of compound 1 and phenalamide A_2_, as well as the spatial relationships observed in the NOESY spectra, the relative stereochemical assignment of the three chiral carbons of compound 1 is tentatively proposed to be 4*R*, 5*R*, and 8*S*. Consequently, the structure of compound 1 was identified and named prophenalamide D.

**Fig. 3:**
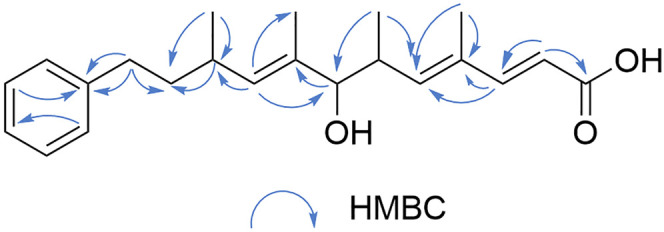
The key HMBC correlations of compounds 2.

Compound 2, acquired as a colorless resinous solid, with the molecular formula C_22_H_30_O_3_, was determined from the HR-ESI-MS ion at *m*/*z* 365.2093 [M + Na]^+^ (*calcd*. 365.2093). The IR spectrum exhibited characteristic absorptions for a carboxylic acid (3409, 1713 and 1623 cm^−1^), a benzene ring (1496, 1454, 747, and 699 cm^−1^).^27^ The ^1^H NMR spectrum (Table 1) revealed a set of signals corresponding to an aldehyde proton [*δ*_H_ 9.43 (s)], five benzene ring protons [*δ*_H_ 7.10–7.28 (5H, m, H-14 to H-18)], three olefinic protons [*δ*_H_ 7.42 (1H, d, *J* = 15.6 Hz, H-3), 5.87(1H, d, *J* = 9.8 Hz, H-5), 5.22 (1H, d, *J* = 9.5 Hz, H-9)], four methyl signals [*δ*_H_ 1.82 (3H, d, *J* = 1.4 Hz, 4-Me), 0.89 (3H, d, *J* = 6.8 Hz, 6-Me), 1.60 (3H, d, *J* = 1.4 Hz, 8-Me), 0.97 (3H, d, *J* = 6.8 Hz, 10-Me)], two methylene signals [*δ*_H_ 1.53–1.70 (2H, m, H-11), 2.51–2.62 (2H, m, H-12)], and three methine signals [*δ*_C_ 2.38–2.47 (1H, m, H-6), 3.77 (1H, d, *J* = 8.1 Hz, H-7), 2.72–2.80 (1H, m, H-10)]. The ^13^C NMR spectrum displayed 22 carbon signals (Table 1), including carboxyl carbon at *δ*_C_ 171.6 (C-1), six benzene carbons [*δ*_C_ 142.6 (C-13), 128.4 (C-14), 128.2 (C-15), 125.6 (C-16), 128.2 (C-17), 128.4 (C-18)], six olefinic carbons [*δ*_C_ 115.2 (C-2), 151.7 (C-3), 133.8 (C-4), 145.7 (C-5), 134.0 (C-8), 135.2 (C-9)], four methyl carbons [*δ*_C_ 16.6 (4-Me), 17.1 (6-Me), 11.6 (8-Me), 21.0 (10-Me)], two methylene carbons [*δ*_C_ 39.0 (C-11), 33.9 (C-12)] and three methine carbons [*δ*_C_ 37.2 (C-6), 82.2 (C-7), 31.9 (C-10)]. The 1D and 2D NMR data (Table 1) indicated that compound 2 shares a similar core structure with compound 1, featuring a polyene side chain connected to a benzene ring. The major structural differences lie in the presence of an additional olefinic double bond in compound 2 and the conversion of the aldehyde group at C-1 in compound 1 to a carboxylic acid in compound 2. The ^1^H NMR signals at *δ*_H_ 5.80 (d, *J* = 15.6 Hz, H-2) and 7.42 (d, *J* = 15.6 Hz, H-3), along with the large coupling constants, indicate the presence of an *E*-olefinic double bond. Notably, the HMBC correlation (Fig. 3) from H-3 to C-4 and C-5 supports the existence of a 2,4-conjugated diene fragment. Additionally, the HMBC correlations from H-2 and H-3 to C-1 (*δ*_C_ 171.60) indicate that this diene moiety is directly attached to a carboxyl group. Furthermore, the HMBC correlation between H-12 and C-13 confirms the attachment of the phenyl ring at C-12. Based on biosynthetic pathway analysis, it is inferred that compound 2 shares the same absolute configuration as compound 1 and phenalamide A_2_,^19^ namely 4*R*, 5*R*, and 8*S* (Fig. S1). Therefore, compound 2 was ultimately identified and named prophenalamide E.

Compound 3 was obtained as a colorless resinous solid. Its molecular formula was as C_33_H_47_NO_4_ based on HR-ESI-MS data, which show a quasi-molecular ion peak at *m/z* 522.3577 [M + H]^+^ (*calcd*. 522.3583). The IR spectrum exhibited diagnostic absorptions for an amino (3350 cm^−1^), a carbonyl or amide group (1728, 1653 and 1535 cm^−1^), a benzene ring (1456 and 775 cm^−1^), suggesting the presence of an amide-bearing benzenoid structure.^27^

**Fig. 4:**
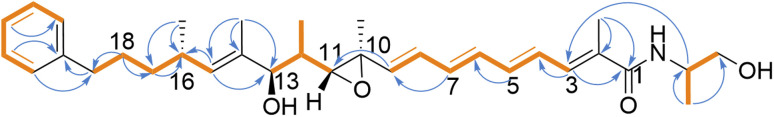
The key HMBC and COSY correlations of compounds 3.

**Fig. 5:**
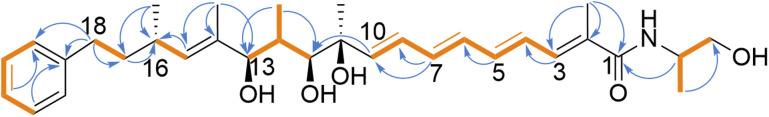
The key HMBC and COSY correlations of compounds 4.

The ^1^H NMR spectrum (Table 2) displayed characteristic signals of five benzene ring protons [*δ*_H_ 7.20 (2H, d, *J* = 7.2 Hz, H-21, 25), 7.23–7.28 (2H, m, H-22, 24), and 7.15 (1H, t, *J* = 7.2 Hz, H-23)], six methyl signals[*δ*_H_ 1.99 (3H, s, 2-Me), 1.35 (3H, s, 10-Me), 0.92 (3H, d, *J* = 6.8 Hz, 12-Me), 1.61 (3H, d, *J* = 1.4 Hz, 14-Me), 0.99 (3H, d, *J* = 6.5 Hz, 16-Me), 1.19 (3H, d, *J* = 6.8 Hz, 1′-Me)], four methylene groups at *δ*_H_ 1.66–1.73 (2H, m, H-17), 2.56–2.70 (4H, m, H-18, 19), and 3.50–3.58 (2H, m, H-2′), five methine protons at *δ*_H_ 3.87 (1H, d, *J* = 4.5 Hz, H-11), 2.26–2.33 (1H, m, H-12), 4.12 (1H, d, *J* = 10.9 Hz, H-13), 2.46–2.51 (1H, m, H-16), and 4.04–4.08 (1H, m, H-1′), and eight olefinic proton signals *δ*_H_ 6.93 (1H, d, *J* = 9.4 Hz, H-3), 6.54–6.61 (2H, m, H-4, 5), 6.38–6.48 (2H, m, H-6, 7, 8), 6.01 (1H, d, *J* = 14.7 Hz, H-9), and 5.30 (1H, d, *J* = 9.3 Hz, H-15). The ^13^C NMR spectra (Table 2) revealed a total of 32 carbon atoms, including one carbonyl carbon at 171.8 (C-1), six benzene carbons [*δ*_C_ 143.9 (C-20), 129.5 (C-21), 129.3 (C-22), 126.7 (C-23), 129.3 (C-24), 129.5 (C-25)], ten olefinic carbons [*δ*_C_ 131.1 (C-2), 135.1 (C-3), 139.1 (C-4), 128.7 (C-5), 129.9 (C-6), 133.2 (C-7), 136.7 (C-8), 139.6 (C-9), 133.6 (C-14), 137.0 (C-15)], six methyls [*δ*_C_ 13.1 (2-Me), 26.9 (10-Me), 10.2 (12-Me), 11.3 (14-Me), 21.5 (16-Me), 17.1 (1′-Me)], four methylenes [*δ*_C_ 40.6 (C-17), 40.8 (C-18), 34.8 (C-19), 66.1 (C-2′)], five methines [*δ*_C_ 87.1 (C-11), 41.6 (C-12), 91.6 (C-13), 33.0 (C-16), 49.0 (C-1′)], and a quaternary carbon [*δ*_C_ 81.5 (C-10)]. A comparison of the 1D and 2D NMR data (Table 2 and Fig. 5) revealed that the structure of compound 3 is closely related to that of the known phenalamid C (5).^19^ The only notable difference observed was the presence of an additional methylene unit [*δ*_H_ 2.55–2.70 (2H, m, H-19)] in the side chain terminating in the benzene ring of compound 3, as indicated by the molecular formula and NMR data. The connectivity of the key structural fragments was established by the ^1^H–^1^H COSY spectrum (Fig. 4), such as H-3/H-4, H-8/H-9, H-12/H-13, and H-15/H-16, H-16/H-17, H-17/H-18, H-18/H-19. Regarding the polyene chain, HMBC correlations from H-3 to C-4, H-5 to C-6, and H-7 to C-8, combined with the extensive olefinic proton signals, confirmed the presence of a conjugated tetraene system (Δ^2,4,6,8^) anchored to the C-1 carbonyl. An all-*E* configuration was assigned to this polyene chain based on the large coupling constants (*J*_8,9_ = 14.7 Hz) and supporting NOESY correlations (Fig. 4).

**Table 2:** ^1^H NMR and ^13^C NMR data of compounds 3 and 4 (in CD_3_OD)^a^

Position	3		Position	4	
	*δ*_C_	*δ*_H_ (*J* in Hz)		*δ*_C_	*δ*_H_ (*J* in Hz)
1	171.8	—	1	171.8	—
2	131.1	—	2	131.3	—
3	135.1	6.93, d (9.4)	3	135.0	6.93, d (9.4)
4	139.1	6.54–6.61, m	4	139.4	6.56–6.59, m
5	128.7	6.54–6.61, m	5	128.9	6.60, d (14.7)
6	129.9	6.38–6.48, m	6	129.0	6.39, d (14.7)
7	133.2	6.38–6.48, m	7	133.3	6.41–6.45, m
8	136.7	6.38–6.48, m	8	136.5	6.41–6.45, m
9	139.6	6.01, d (14.7)	9	142.1	5.97, d (14.7)
10	81.5	—	10	83.7	—
11	87.1	3.87, d (4.5)	11	85.1	3.66, d (9.4)
12	41.6	2.26–2.33, m	12	43.7	2.00–2.04, m
13	91.6	4.12, d (10.9)	13	89.4	3.86, d (9.8)
14	133.6	—	14	133.6	—
15	137.0	5.30, d (9.3)	15	137.0	5.31, d (9.3)
16	33.0	2.46–2.51, m	16	33.0	2.43–2.50, m
17a	40.6	1.66–1.73, m	17a	40.5	1.54–1.60, m
17b		1.66–1.73, m	17b		1.65–1.72, m
18a	40.8	2.64–2.70, m	*	—	—
18b		2.64–2.70, m			
19a	34.8	2.64–2.70, m	18a	34.8	2.64–2.69, m
19b		2.56–2.62, m	18b		2.54–2.61, m
20	143.9	—	19	143.9	—
21	129.5	7.20, d (7.2)	20	129.4	7.19, d (7.2)
22	129.3	7.23–7.28, m	21	129.3	7.23–7.28, m
23	126.7	7.15, t (7.2)	22	126.6	7.15, t (7.2)
24	129.3	7.23–7.28, m	23	129.3	7.23–7.28, m
25	129.5	7.20, d (7.2)	24	129.4	7.19, d (7.2)
2-Me	13.1	1.99, d (1.4)	2-Me	13.1	1.99, s
10-Me	26.9	1.35, s	10-Me	22.3	1.29, s
12-Me	10.2	0.92, d (6.8)	12-Me	13.9	0.99, d (6.5)
14-Me	11.2	1.61, d (1.4)	14-Me	11.3	1.63, s
16-Me	21.5	0.99, d (6.8)	16-Me	21.4	0.98, d (6.5)
1′-Me	17.1	1.20, d (6.8)	1′-Me	17.1	1.19, d (6.8)
1′	49.0	4.04–4.08, m	1′	49.8	4.04–4.09, m
2′	66.1	3.50–3.58, m	2′	66.1	3.50–3.58, m

a ^1^H NMR spectra measured at 600 MHz, ^13^C NMR spectra measured at 150 MHz. ‘*’ represents that the space left out corresponds to other similar signals of another compound.

By comparing the NMR data with literature values and considering their biogenetic relationship,^19,28,29^ the absolute configuration of compound 3 was established as 12*R*, 13*R*, 16*S*. This assignment relies on the biogenetic consistency typically observed in myxobacterial secondary metabolites, where structurally related compounds originating from conserved biosynthetic pathways maintain stereochemical uniformity.^19,28,29^

Given that the epoxide ring imparts a rigid conformational framework to this local region, precedents in the literature have successfully determined the configuration of the epoxide moiety using NOE analysis.^30^ Within this conformationally constrained system, the coupling constant *J*_11,12_ = 4.5 Hz and the distinct H-11/H-12 NOESY correlation confirmed a *gauche* conformation between these two protons in compound 3.^31^ Based on this spatial arrangement and the defined 12*R* configuration, the relative configuration of C-11 was unambiguously assigned as 11R*. Subsequently, a key NOESY correlation between 10-Me and 12-Me, evaluated within the context of the 11R*, 12*R* stereocenters, allowed the relative configuration at C-10 to be deduced as 10S*. Consequently, based on the comprehensive spectroscopic analysis and biogenetic analysis, compound 3 was assigned as 10S*, 11R*, 12*R*, 13*R*, 16*S* and named phenalamide F.

Compound 4 was obtained as a colorless resinous solid. Its molecular formula was determined to be C_32_H_47_NO_5_ based on HR-ESI-MS data, which showed ion peaks at *m*/*z* 564.3087 [M + K]^+^ (*calcd*. 564.3091). The IR spectrum exhibited characteristic absorptions for an amino (3380 cm^−1^), a carbonyl (1728, 1636 and 1521 cm^−1^), a benzene ring (1454 and 771 cm^−1^).^27^ The ^1^H NMR spectrum (Table 2) displayed characteristic signals of five benzene ring protons [*δ*_H_ 7.19 (2H, d, *J* = 7.2 Hz, H-20, 24), 7.23–7.28 (2H, m, H-21, 23), and 7.15 (1H, t, *J* = 7.2 Hz, H-22)], six methyl signals[*δ*_H_ 1.99 (3H, s, 2-Me), 1.29 (3H, s, 10-Me), 0.99 (3H, d, *J* = 6.5 Hz, 12-Me), 1.63 (3H, s, 14-Me), 0.98 (3H, d, *J* = 6.5 Hz, 16-Me), 1.19 (3H, d, *J* = 6.8 Hz, 1′-Me)], three methylene groups at *δ*_H_ 1.54–1.72 (2H, m, H-17), 2.54–2.69 (2H, m, H-18), and 3.50–3.58 (2H, m, H-2′), five methine protons at *δ*_H_ 3.66 (1H, d, *J* = 9.4 Hz, H-11), 2.00–2.04 (1H, m, H-12), 3.86 (1H, d, *J* = 9.8 Hz, H-13), 2.43–2.50 (1H, m, H-16), and 4.04–4.09 (1H, m, H-1′), and eight olefinic proton signals *δ*_H_ 6.93 (1H, d, *J* = 9.4 Hz, H-3), 6.56–6.59 (1H, m, H-4), 6.60 (1H, d, *J* = 14.7 Hz, H-5), 6.39 (1H, d, *J* = 14.7 Hz, H-6), 6.41–6.45 (1H, m, 2H, H-7, 8), 5.97 (1H, d, *J* = 14.7 Hz, H-9), and 5.31 (1H, d, *J* = 9.3 Hz, H-15). The ^13^C NMR spectra (Table 2) revealed a total of 32 carbon atoms, including one carbonyl carbon at 171.8 (C-1), six benzene carbons [*δ*_C_ 143.9 (C-19), 129.4 (C-20), 129.3 (C-21), 126.6 (C-22), 129.3 (C-23), 129.4 (C-24)], ten olefinic carbons [*δ*_C_ 131.3 (C-2), 135.0 (C-3), 139.4 (C-4), 128.9 (C-5), 129.0 (C-6), 133.3 (C-7), 136.5 (C-8), 142.1 (C-9), 133.6 (C-14), 137.0 (C-15)], six methyls [*δ*_C_ 13.1 (2-Me), 22.3 (10-Me), 13.9 (12-Me), 11.3 (14-Me), 21.4 (16-Me), 17.1 (1′-Me)], three methylenes [*δ*_C_ 40.5 (C-17), 34.8 (C-18), 66.8 (C-2′)], five methines [*δ*_C_ 85.1 (C-11), 43.7 (C-12), 89.4 (C-13), 33.0 (C-16), 49.8 (C-1′)], and a quaternary carbon [*δ*_C_ 83.7 (C-10)]. Compound 4 possesses a planar structure closely related to phenalamide A_2_,^16^ distinguished primarily by the oxidation of the C-10/C-11 fragment. NMR analysis further confirmed that the Δ^10^ olefinic unsaturation in phenalamide A_2_ has been replaced by a vicinal diol functionality in compound 4, as evidenced by the diagnostic upfield shifts of the C-10 and C-11 signals [*δ*_C_ 83.7 (C-10), 85.1 (C-11)], which are characteristic of the oxymethines in the 10,11-diol system. Given the highly conserved biosynthetic substitution pattern within this class of compounds,^19,28,29^ a comprehensive 1D and 2D NMR comparison with phenalamide A2, 3, and 5 initially allowed the absolute configuration of 4 to be assigned as 12*R*, 13*R*, 16*S*.

Mechanistically, the epoxidation of a vicinal diol proceeds *via* mono-activation followed by a stereospecific intramolecular SN_2_ displacement.^31–34^ This cyclization inherently dictates strict Walden inversion at the electrophilic carbon and retention at the nucleophilic oxygen.^31–34^ In this study, the structure of 4 differs from 3 primarily by the presence of an acyclic C-10/C-11 vicinal diol rather than an epoxide ring. Within this mechanistic context, compound 3 is postulated to be the biosynthetic cyclization product of the diol precursor 4. To unambiguously determine the regioselectivity of this nucleophilic attack (*i.e.*, whether C-10 or C-11 acts as the electrophile), comparative NMR data were rigorously analyzed. The coupling constant shifted dramatically from a small *J*_11,12_ = 4.5 Hz in the rigid epoxide 3 to a large *J*_11,12_ = 9.4 Hz in the acyclic diol 4, strongly indicating a highly preferred *anti*-periplanar conformation^31^ between H-11 and H-12 in the latter. Despite the inherent conformational flexibility of the acyclic diol moiety, this large *J*_11,12_ coupling, combined with the diagnostic 10-Me/12-Me NOESY correlation and the previously established 12*R* stereocenter, securely restricts the relative configuration at C-11 in 4 to be 11S*.

The establishment of an 11S* geometry in 4, contrasting with the 11R* configuration in the rigid epoxide 3, provides compelling evidence for a strict Walden inversion during the epoxidation event Fig. 5. This stereochemical outcome mechanistically dictates that the 10-OH group executes a backside nucleophilic attack on the activated C-11. Because the oxygen atom serves as the nucleophile, the C–O bond at C-10 remains intact, allowing this stereocenter to inherently retain its configuration as 10S*.

Integrating this robust spectroscopic evidence with the mechanistic rationale, the configuration of the diol fragment in 4 was unequivocally deduced as 10S*, 11S*, 12*R*, 13*R*, 16*S*. Consequently, compound 4 was identified and designated as phenalamide G.

### 2.2 Inhibitory effects on spore germination and mycelial growth against *Fusarium sacchari*

**Fig. 6:**
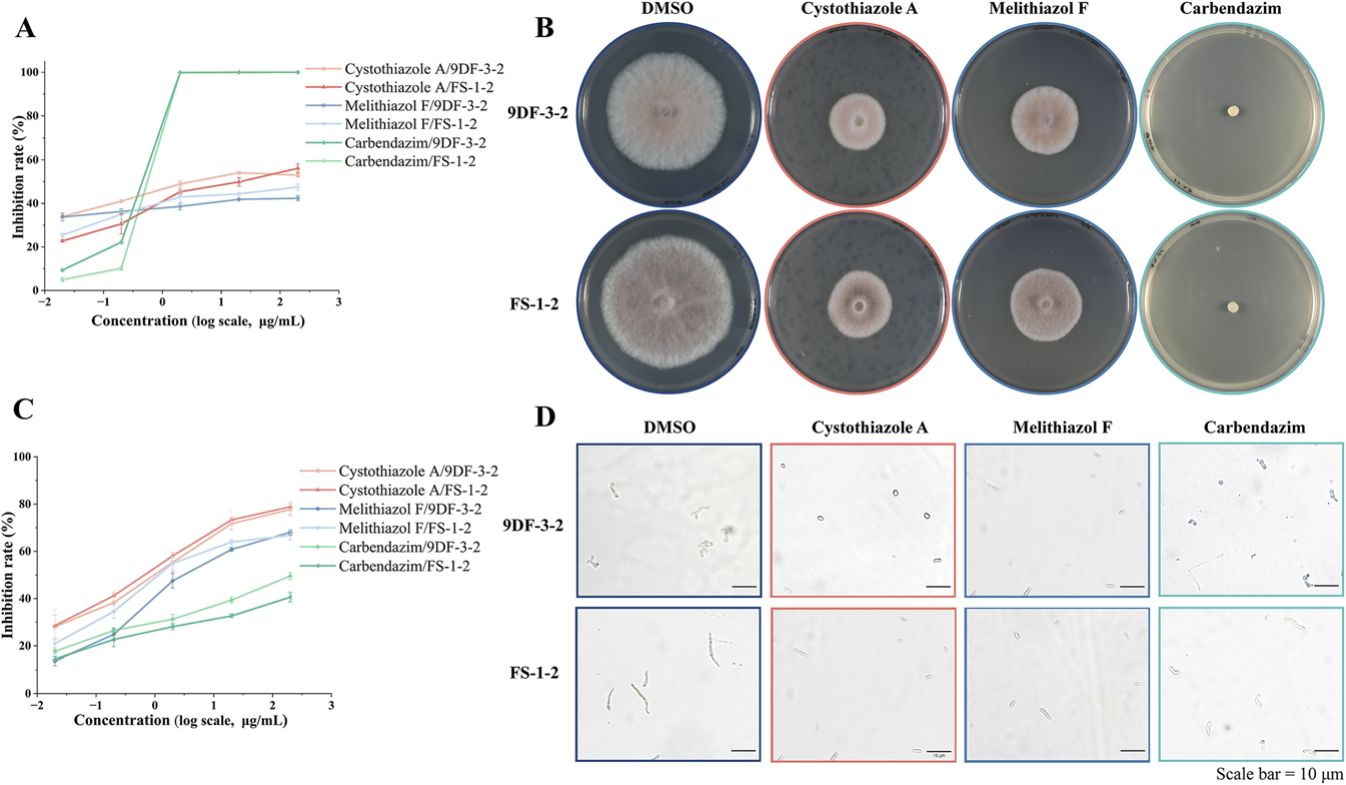
Inhibitory effects of Cystothiazole A and Melithiazol F on *Fusarium sacchari* 9DF-3-2 and FS-1-2. (A) Mycelial growth inhibition assay; (B) plate culture illustrating the suppression of mycelial growth (diameter of the plate = 90 mm) at the concentration of 100 µg mL; (C) inhibitory effects on spore germination; (D) microscopic images demonstrating spore germination inhibition at the concentration of 100 µg mL^−1^ (*n* = 3).

In our study, all the isolated compounds were evaluated for antifungal activity against *F. sacchari*, and notably, only cystothiazole A and melithiazol F had significant inhibitory effects. Fungal spore dispersal plays an important role in the spread of sugarcane Pokkah boeng disease. Nevertheless, most antifungal studies on *F. sacchari* have focused on mycelial growth inhibition, and comparatively little attention has been paid to spore germination.^15–17^ In the present study, cystothiazole A not only inhibits the mycelial growth of *F*. *sacchari* strains 9DF-3-2 and FS-1-2, but also strongly suppresses spore germination (Fig. 6).

In the mycelial growth inhibition assay (Table 3), the EC_50_ values of cystothiazole A against *F. sacchari* 9DF-3-2 and FS-1-2 were 3.01 µg mL^−1^ and 9.71 µg mL^−1^, respectively. At lower concentrations, both cystothiazole A and melithiazol F exhibited more potent inhibition than carbendazim. In the spore germination inhibition assay (Table 3), cystothiazole A demonstrated the most powerful effect, with EC_50_ values of 1 µg mL^−1^ for *F. sacchari* 9DF-3-2 and 0.67 µg mL^−1^ for FS-1-2, significantly outperforming carbendazim across all tested concentrations. The EC_50_ values of melithiazol F were 2.94 µg mL^−1^ and 1.08 µg mL^−1^, respectively. While its effect was comparable to that of carbendazim at lower concentrations, it was significantly stronger at higher concentrations. Additionally, microscopic observations (Fig. 6) revealed that spores treated with DMSO exhibited swelling, radial elongation, and gradual germ tube extension. In contrast, spores treated with cystothiazole A and melithiazol F remained in an ovoid dormant state, showing no signs of germination.

**Table 3:** Inhibition effect on mycelial growth and spore germination of *Fusarium sacchari* 9DF-3-2 and FS-1-2^a^

Compounds	Spore germination		Mycelial growth	
	9DF-3-2	FS-1-2	9DF-3-2	FS-1-2
Cystothiazole A	1.00 ± 0.23	0.67 ± 0.34	3.01 ± 0.20	9.71 ± 1.22
Melithiazol F	2.94 ± 0.45	1.08 ± 0.35	>200	>200
Carbendazim	>200	>200	0.31 ± 0.14	0.38 ± 0.17

a Results are expressed as EC_50_ values in µg mL^−1^. Carbendazim was used as positive control (*n* = 3, with 100 spores observed per replicate).

### 2.3 Assessment of the compromise of the *Fusarium sacchari* spore membrane by nucleic acid and protein leakage

**Fig. 7:**
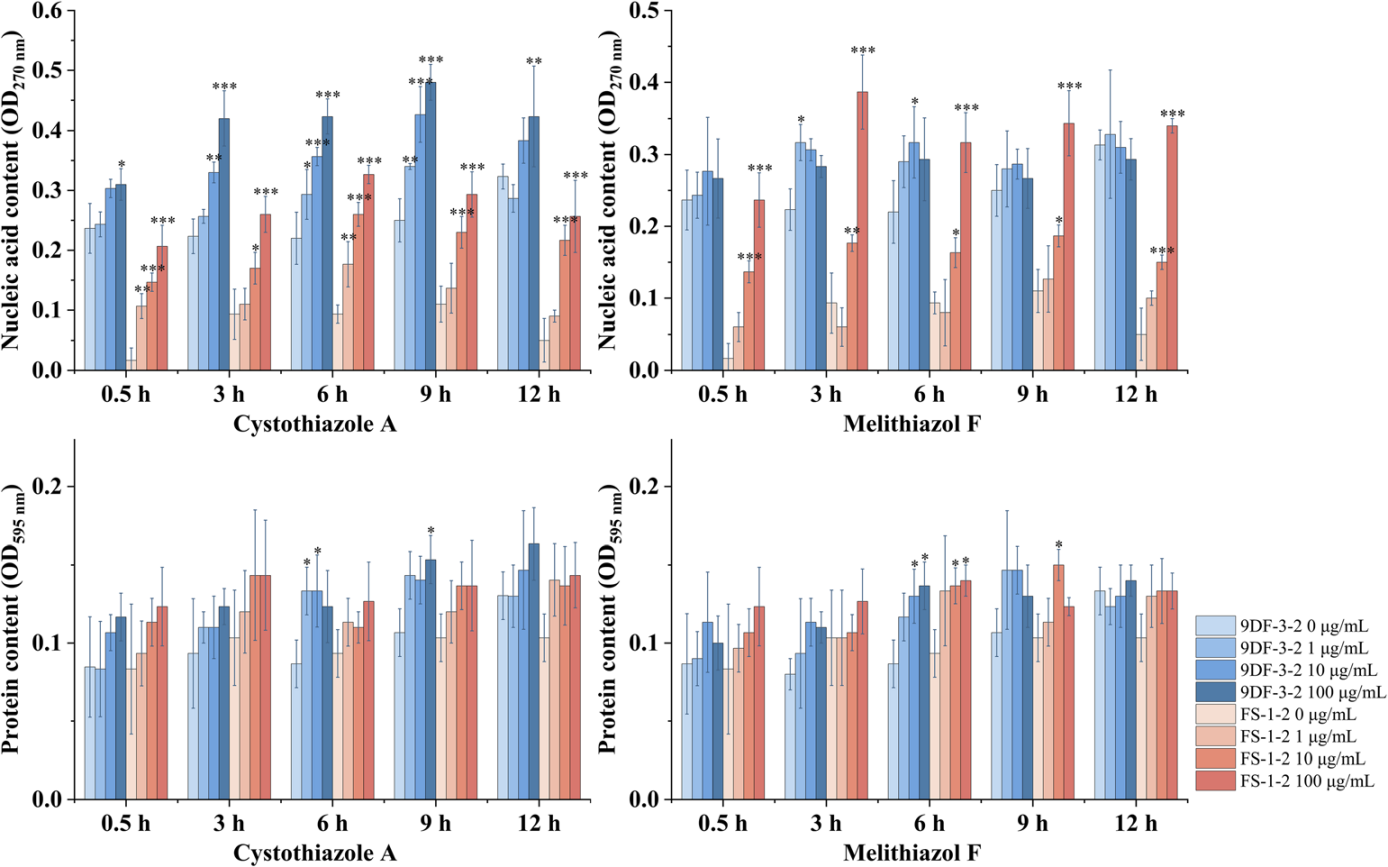
Extracellular nucleic acid and protein levels in *Fusarium sacchari* spores treated with varying concentrations of Cystothiazole A and Melithiazol F. Statistical significance compared to the 0 µg mL^−1^ group is indicated as follows: *p* < 0.05 (*), *p* < 0.01 (**), *p* < 0.001 (***), and *p* > 0.05 (unlabeled) (*n* = 3).

Cell membrane permeability can be evaluated by measuring intracellular leakage, with nucleic acid absorbance at 260 nm indicating membrane integrity and Coomassie Brilliant Blue G-250 binding at 595 nm quantifying protein content.^35^ This study assessed nucleic acid leakage from *Fusarium sacchari* spores following exposure to varying concentrations of cystothiazole A (7) and melithiazol F (9) over different incubation periods (Fig. 7).

The results indicate that cystothiazole A induced a significant, concentration-dependent increase in nucleic acid leakage in both *F. sacchari* strains. While the effect was negligible at 1 µg mL^−1^ compared to the negative control, treatment at 10 µg mL^−1^ and 100 µg mL^−1^ resulted in a marked increase in leakage, reaching maximum absorbance values of 0.48 for strain 9DF-3-2 and 0.38 for strain FS-1-2 at the highest concentration. In contrast, melithiazol F exhibited strain-specific effects. It showed minimal impact on nucleic acid leakage in strain 9DF-3-2 but induced a concentration-dependent increase in strain FS-1-2, with a peak absorbance of 0.39 at 100 µg mL^−1^.

Regarding protein leakage, both compounds showed moderate effects (Fig. 7). In all treated groups, protein leakage increased slightly, but the change was not statistically significant. This differential leakage pattern, significant release of nucleic acids (absorbing at 260 nm) with limited protein efflux, suggests that these compounds may compromise membrane barrier function selectively or partially, rather than causing complete cell lysis. Such an effect could be attributed to alterations in membrane permeability, the formation of specific pores, or localized membrane damage, which permits the efflux of certain intracellular materials while retaining larger protein complexes, or potentially reflects the activation of fungal stress response and repair mechanisms.

### 2.4 Biological evaluation and structure–activity relationship discussion

Among all isolated compounds, cystothiazole A (7) and melithiazol F (9) showed the strongest antifungal activity against *Fusarium sacchari*, whereas the newly identified phenalamide analogues (1–4) were inactive under the tested conditions. This result suggests that the antifungal activity of *Myxococcus stipitatus* GXUA 01510 against *F. sacchari* is not broadly associated with all secondary metabolites produced by the strain, but is mainly attributable to specific active constituents. In particular, the lack of activity of compounds 1–4 indicates that the phenalamide scaffold identified here does not inherently confer inhibitory activity against *F. sacchari*, at least under our assay conditions. By contrast, the thiazole-containing metabolites 7 and 9 were both relatively abundant in the extract and biologically active, supporting their role as major contributors to the antifungal activity of GXUA 01510. Moreover, the inhibition of spore germination and mycelial growth, together with the leakage of nucleic acids and proteins, provides preliminary evidence that these active metabolites may impair membrane integrity.^36^ Therefore, our results not only define the principal antifungal constituents of GXUA 01510 but also provide a basis for further evaluation of thiazole-containing myxobacterial metabolites as candidates for the control of sugarcane Pokkah boeng disease.

## 3 Experimental section

### 3.1 General experimental procedures

^1^H-NMR and ^13^C-NMR spectra were recorded in CDCl_3_ or CD_3_OD using AVIII HD 600 (Bruker, Billerica) and AVIII 500 NMR spectrometers (Bruker, Switzerland). The 600 Hz NMR spectra in the research were collected by the NMR laboratory of School of Chemistry and Chemical Engineering, Guangxi University. HR-ESI-MS spectra were obtained using a Xevo® G2-S UPLC-QTOF mass spectrometer (Waters, USA). A Shimadzu LC-2030C 3D Plus high-performance liquid chromatograph (HPLC) (Shimadzu, Japan) was used for compound isolation and purification. The stationary phase for reversed-phase chromatography was ODS (C18 MB100-40/75, Fuji Chemical Industries). The analytical HPLC column used was a YMC-Pack ODS-A column (250 mm × 4.6 mm, 5 µm), while the semi-preparative HPLC column used was a YMC-Pack ODS-A column (250 mm × 10 mm, 5 µm). Silica gel GF254 plates (Qingdao Puke Separation Materials Co., Ltd) were used for preparative thin-layer chromatography (TLC). Optical rotations were measured on an SGW-1 automatic polarimeter (Shanghai Wuguang, China).

### 3.2 Fungal and bacterial strain material

The strain *Myxococcus stipitatus* GXUA 01510 was isolated from the Beibu Gulf, Guangxi, China, and its genome was sequenced and identified by Professor Zhiwei Su. The strain is currently preserved at the College of Agriculture, Guangxi University. For antimicrobial activity assays, *Fusarium sacchari*, the predominant pathogen responsible for sugarcane Pokkah boeng disease in Guangxi, was used as the test strain. Two isolates with varying pathogenicity, 9DF-3-2 and FS-1-2, were selected, with FS-1-2 exhibiting higher virulence than 9DF-3-2. These strains were isolated, identified, and provided by Professor Chengwu Zou from the Guangxi Key Laboratory of Sugarcane Biology, Guangxi University.

### 3.3 Extraction and isolation

The strain *Myxococcus stipitatus* GXUA 01510 was inoculated into VY/2 medium for large-scale cultivation at 30 °C for four days. A soybean-sized portion of the mycelium was then transferred to IR1 medium and incubated at 30 °C for six days, resulting in a total fermentation of 2500 IR1 plates (approximately 75 L medium). The mycelium was collected from the IR1 medium and extracted with acetone until the solution became colorless. The combined extracts were concentrated under reduced pressure to yield approximately 20 g of crude extract. This mixture was subjected to gradient elution using hexane–acetone (100 : 0 to 50 : 50, v/v), followed by a methanol wash. Thin-layer chromatography (TLC) analysis resulted in nine fractions, designated Ms-1 to Ms-9.

Fraction Ms-3 (0.63 g) was dissolved in chloroform containing a small amount of methanol and concentrated under reduced pressure to near saturation, allowing crystals to form. The crystals were collected by filtration and purified by HPLC (acetonitrile–water, 80 : 20, v/v) to yield compounds 7 (88.8 mg, 0.444% yield) and 8 (2.4 mg, 0.012% yield).

Fraction Ms-4 (0.26 g) was separated by HPLC (ODS, acetonitrile-water 75 : 25), yielding subfractions Ms-4-1 to Ms-4-5. Subfractions Ms-4-4 and Ms-4-5 were further purified by preparative TLC (petroleum ether: acetone: methanol 95 : 5 : 1), resulting in compounds 9 (134.6 mg, 0.673% yield) and 6 (3.2 mg, 0.016% yield).

Fraction Ms-5 (1.08 g) was fractionated using MPLC (ODS, methanol–water 50 : 50 to 100 : 0), yielding subfractions Ms-5-1 to Ms-5-8. Subfraction Ms-5-4 (248.3 mg) was further separated by HPLC (ODS, acetonitrile-water = 70 : 30) and purified by preparative TLC (dichloromethane: methanol = 98 : 2) to yield compounds 1 (15.0 mg, 0.075% yield) and 2 (4.33 mg, 0.022% yield).

Fraction Ms-6 (3.35 g) was separated using MPLC (ODS, acetonitrile-water 50 : 50 to 100 : 0), yielding subfractions Ms-6-1 to Ms-6-9. Subfraction Ms-6-4 (590.0 mg) was further purified by HPLC (ODS, acetonitrile-water 65 : 35) to obtain compounds 3 (7.8 mg, 0.039% yield), 4 (7.5 mg, 0.038% yield), and 5 (15.0 mg, 0.075% yield).

Fraction Ms-8 (3.01 g) was dissolved in methanol and filtered to obtain Ms-8M, which was then fractionated using MPLC (ODS, methanol–water 5 : 95 to 40 : 60) into subfractions Ms-8-1 to Ms-8-11. Subfraction Ms-8-5 was further separated by HPLC (ODS, acetonitrile-water 70 : 30) into subfractions Ms-8-5-1 to Ms-8-5-6. Subfraction Ms-8-5-1 (34.3 mg) was purified by recrystallization: the sample was dissolved in methanol, concentrated under reduced pressure to near saturation, allowed to crystallize, filtered, and washed with a small amount of methanol to yield compound 10 (13 mg, 0.065% yield). Subfraction Ms-8-5-3 was identified as compound 12 (13.8 mg, 0.069% yield). Subfractions Ms-8-5-5 (23.2 mg) and Ms-8-5-4 (13.0 mg) were purified by preparative TLC (dichloromethane: methanol 9 : 1) to obtain compounds 11 (19.1 mg, 0.096% yield) and 13 (6.5 mg, 0.033% yield), respectively.

The isolation yields for the major metabolites (5, 7, 10) were satisfactory, reflecting their high abundance in the crude extract. In contrast, the minor compounds (1–4, 6, 8, 9) were obtained in low yields, a common challenge attributed to their low natural titers and multi-step purification losses. Nevertheless, these yields were deemed sufficient for the primary objective of this study, which was the complete structural elucidation and characterization of the isolates. While future optimization of fermentation and purification could enhance yields, the current discovery-oriented approach proved effective.

#### 3.3.1 Prophenalamide D (1)

Colorless resinous solid; [α]^25^_D_ + 63.1 (*c* 0.02, MeOH); UV *λ*_max_ (Δ*ε*) 290 nm (3.21); IR (KBr) *ν*_max_ 3026, 2956, 2925, 2866, 1675, 1639, 1603, 1496, 1453, 810, 746, 699 cm^−1^; For ^1^H NMR (CD_3_OD, 500 MHz) and ^13^C NMR (CD_3_OD, 125 MHz) spectroscopic data, see Table 1, HR-ESI-MS *m*/*z*: 365.2093 [M + Na]^+^ (*calcd*. 365.2093).

#### 3.3.2 Prophenalamide E (2)

Colorless resinous solid; [α]^25^_D_ + 64.5 (*c* 0.03, MeOH); UV *λ*_max_ (Δ*ε*) 290 nm (3.44); IR (KBr) *ν*_max_ 3409, 2927, 2856, 1713, 1623, 1496, 1454, 970, 950, 747, 699 cm^−1^; For ^1^H NMR (CD_3_OD, 500 MHz) and ^13^C NMR (CD_3_OD, 125 MHz) spectroscopic data, see Table 1, HR-ESI-MS *m*/*z*: 365.2093 [M + Na]^+^ (*calcd*. 365.2093).

#### 3.3.3 Phenalamide F (3)

Colorless resinous solid; [α]^25^_D_ + 23.3 (*c* 0.05, MeOH); UV *λ*_max_ (Δ*ε*) 328 nm (4.49); IR (KBr) *ν*_max_ 3330, 2957, 2931, 2876, 1632, 1520, 1452, 1279, 1165, 999, 750, 700 cm^−1^; For ^1^H NMR (CD_3_OD, 600 MHz) and ^13^C NMR (CD_3_OD, 150 MHz) spectroscopic data, see Table 2, HR-ESI-MS *m*/*z*: 522.3577 [M + H]^+^ (*calcd*. 522.3583).

#### 3.3.4 Phenalamide G (4)

Colorless resinous solid; [α]^25^_D_ + 54.2 (*c* 0.02, MeOH); UV *λ*_max_ (Δ*ε*) 328 nm (4.40); IR (KBr) *ν*_max_ 3380, 2959, 2930, 2867, 1728, 1636, 1521, 1454, 1260, 1222, 999, 771 cm^−1^; For ^1^H NMR (CD_3_OD, 600 MHz) and ^13^C NMR (CD_3_OD, 150 MHz) spectroscopic data, see Table 2, HR-ESI-MS *m*/*z*: 564.3087 [M + K]^+^ (*calcd*. 564.3091).

### 3.4 Spore germination inhibition assay

*Fusarium sacchari* strains 9DF-3-2 and FS-1-2 were cultured on PDA for seven days. The spores were collected by rinsing plates with sterile water, filtered through double-layer gauze, centrifuged (2000 rpm, 1 min), and resuspended in sterile water to obtain a concentration of 1 × 10^6^ spores per mL. Cystothiazole A and melithiazol F stock solutions (0.002–20 mg mL^−1^ in DMSO) were added to PDB containing 1 × 10^5^ spores per mL at a 1 : 100 ratio, yielding final concentrations of 0.02–200 µg mL^−1^. Cultures were incubated at 28 °C and 180 rpm for 8 h. Germination was assessed microscopically, with a spore defined as germinated when the germ tube length exceeded its short-axis diameter. At least 100 spores were counted in triplicate per treatment. DMSO and carbendazim served as negative and positive controls, respectively.

### 3.5 Mycelial growth inhibition assay

Cystothiazole A and melithiazol F stock solutions (0.002–20 mg mL^−1^ in DMSO) were incorporated into molten PDA at a 1 : 100 ratio to obtain final concentrations of 0.02–200 µg mL^−1^. The medium was poured into plates and allowed to solidify. A 5 mm *Fusarium sacchari* mycelial plug, excised from the margin of a 7 day-old colony, was placed inverted at the center of each plate. Carbendazim and DMSO served as positive and negative controls, respectively. The treatments were performed in triplicate, and the plates were incubated at 26 °C for five days. Colony diameters for each treatment were measured using the cross-intersection method, and the inhibition rate was calculated as the percentage reduction in colony diameter of the treatment group relative to the negative control, with 0.5 cm (the diameter of the inoculated mycelia) subtracted from both measurements.

### 3.6 Assays for nucleic acid and protein leakage from spores

The absorbance at 260 nm and 595 nm was measured to determine the nucleic acid and protein concentrations in the supernatant, respectively. Increases indicate disruption of spore cell membrane integrity and leakage of intracellular contents. Soybean-sized mycelial plugs of *Fusarium sacchari* 9DF-3-2 and FS-1-2 were grown on PDA for 7 days, inoculated into 100 mL PDB, and incubated at 26 °C for 7 days. The culture broth was centrifuged at 4500 rpm for 3 min, and the supernatant was centrifuged again at 10 000 rpm for 2 min. Spores in the remaining supernatant were resuspended in sterile PBS and adjusted to 10^7^–10^8^ spores per mL. A 12 mL aliquot was mixed with 120 µL of compound stock solution to final concentrations of 1, 10, and 100 µg mL^−1^. At 0, 3, 6, 9, and 12 h, 2 mL samples were collected and centrifuged (10 000 rpm, 2 min), and the supernatant was used for leakage assays. Pellets were used for microscopic observation. Coomassie Brilliant Blue G-250 reagent was prepared by dissolving 20 mg of dye in 10 mL of 95% ethanol, adding 24 mL of 85% phosphoric acid, and diluting to 200 mL with water. For nucleic acids, 1 mL of the supernatant was read at 260 nm; for proteins, 1 mL was mixed with 5 mL of dye reagent and read at 595 nm after 2–5 min. DMSO served as the negative control, and all the treatments were performed in triplicate.

## 4 Conclusions

In this study, the chemical exploration of *Myxococcus stipitatus* GXUA 01510 led to the discovery of prophenalamides D-E (1–2) and phenalamides F-G (3–4), a new series of phenyl polyenes that expands the structural diversity of myxobacterial secondary metabolites. Notably, the isolation of compounds 10–13, previously unknown in myxobacteria, highlights the untapped biosynthetic potential of this genus. The biological evaluation revealed thiazole-containing metabolites, cystothiazole A (7) and melithiazol F (9), as potent antifungals against *Fusarium sacchari*. Cystothiazole A uniquely suppresses spore germination, a rare trait among natural antifungals that usually target mycelial growth. The observed leakage of intracellular macromolecules further suggests that these compounds function by compromising cell membrane integrity. Overall, these findings help clarify the chemical basis underlying the antifungal activity of GXUA 01510 and provide a foundation for further evaluation of myxobacterial metabolites for the management of sugarcane Pokkah boeng disease.

## Author contributions

Zhi-Wei Su and Zhong-Hui Ma conceptualized the projects, revised the manuscript, and provided financial support. Wang Jiang, Qun-Hao Li, Ahmed F. Elkarmout, and Jia-Song Guan implemented the studies and wrote the manuscript. All authors approved the final version of the manuscript.

## Conflicts of interest

There are no conflicts to declare.

## Supplementary Material

RA-016-D5RA10061E-s001

## Data Availability

The data supporting this article have been included as part of the supplementary information (SI). Supplementary information is available. See DOI: https://doi.org/10.1039/d5ra10061e.
