# Supplementary material for: Antifungal metabolites from Myxococcus stipitatus GXUA 01510 for the control of sugarcane Pokkah boeng disease caused by Fusarium sacchari
Source: RSC Adv. 2026 May 6;16(26):23544–53. doi: 10.1039/d5ra10061e (PMC13148359; doi:10.1039/d5ra10061e)
Supplement: RA-016-D5RA10061E-s001 [file RA-016-D5RA10061E-s001.pdf]

Supplementary Information (SI) for RSC Advances.
This journal is © The Royal Society of Chemistry 2026

Supporting Information

Antifungal metabolites from *Myxococcus stipitatus* GXUA 01510 for the control of sugarcane Pokkah boeng disease caused by *Fusarium sacchari*

Wang Jiang,[a] Qun Hao Li,[a] Jiasong Guan,[a] Ahmed F. Elkarmout,[a,b] Zhonghui Ma*[a] and Zhiwei Su*[a]

[a] National Demonstration Center for Experimental Plant Science Education, College of Agriculture, Guangxi University, Nanning 530004, Guangxi, China.

[b] Department of Horticulture, Faculty of Agriculture, Tanta University, Tanta, Egypt.

* Correspondence.

Zhonghui Ma. E-mail: mazhonghui@gxu.edu.cn

Zhiwei Su. E-mail: suzhiwei@gxu.edu.cn

Table of Contents

Table S1. ^{1}H NMR and ^{13}C NMR data of compounds **1** (in $CDCl_3$) **3** and phenalamide A_2[1] (in CD_3OD)

Figure S1 Predicted biosynthetic structure of phenalamide

Figure S2 HRESI-MS spectrum of phenalamide D (**1**)

Figure S3 ^{1}H NMR spectrum of phenalamide D (**1**) in $CDCl_3$ (500 MHz)

Figure S4 ^{13}C NMR spectrum of phenalamide D (**1**) in $CDCl_3$ (125 MHz)

Figure S5 HSQC spectrum of phenalamide D (**1**) in $CDCl_3$

Figure S6 HMBC spectrum of phenalamide D (**1**) in $CDCl_3$

Figure S7 ^{1}H-^{1}H COSY spectrum of phenalamide D (**1**) in $CDCl_3$

Figure S8 NOESY spectrum of phenalamide D (**1**) in $CDCl_3$

Figure S9 IR spectrum of phenalamide D (**1**)

Figure S10 HRESI-MS spectrum of phenalamide E (**2**)

Figure S11 ^{1}H NMR spectrum of phenalamide E (**2**) in $CDCl_3$ (500 MHz)

Figure S12 ^{13}C NMR spectrum of phenalamide E (**2**) in $CDCl_3$ (125 MHz)

Figure S13 HSQC spectrum of phenalamide E (**2**) in $CDCl_3$

Figure S14 HMBC spectrum of phenalamide E (**2**) in $CDCl_3$

Figure S15 IR spectrum of phenalamide E (**2**)

Figure S16 HRESI-MS spectrum of phenalamide F (**3**)

Figure S17 ^{1}H NMR spectrum of phenalamide F (**3**) in CD_3OD (600 MHz)

Figure S18 ^{13}C NMR spectrum of phenalamide F (**3**) in CD_3OD (150 MHz)

Figure S19 HSQC spectrum of phenalamide F (**3**) in CD_3OD

Figure S20 HMBC spectrum of phenalamide F (**3**) in CD_3OD

Figure S21 ^{1}H-^{1}H COSY spectrum of phenalamide F (**3**) in CD_3OD

Figure S22 NOESY spectrum of phenalamide F (**3**) in CD_3OD

Figure S23 IR spectrum of phenalamide F (**3**)

Figure S24 HRESI-MS spectrum of phenalamide G (**4**)

Figure S25 ^1H NMR spectrum of phenalamide G (**4**) in CD_3OD (600 MHz)

Figure S26 ^{13}C NMR spectrum of phenalamide G (**4**) in CD_3OD (150 MHz)

Figure S27 HSQC spectrum of phenalamide G (**4**) in CD_3OD

Figure S28 HMBC spectrum of phenalamide G (**4**) in CD_3OD

Figure S29 ^1H-^1H COSY spectrum of phenalamide G (**4**) in CD_3OD

Figure S30 NOESY spectrum of phenalamide G (**4**) in CD_3OD

Figure S31 IR spectrum of phenalamide G (**4**)

Figure S32. HPLC purity chromatograms of the isolated new compounds and potent active.

Table S1. ^{1}H NMR and ^{13}C NMR data of compounds **1** (in $CDCl_3$), **3** and phenalamide A_2[1] (in CD_3OD).

Position	**1**			**3**			**phenalamide A_2**	
	δ_C	δ_H (*J* in Hz)	Position	δ_C	δ_H (*J* in Hz)	Position	δ_C	δ_H (*J* in Hz)
*	—	—	1	171.8	—	1	171.8	—
*	—	—	2	131.3	—	2	130.6	—
*	—	—	3	135.0	6.93, d (9.4)	3	135.2	6.96, d (9.1)
*	—	—	4	139.4	6.56-6.59, m	4	128.4	6.58, m
*	—	—	5	128.9	6.60, d (14.7)	5	139.9	6.58, m
*	—	—	6	129.0	6.39, d (14.7)	6	127.6	6.40, m
*	—	—	7	133.3	6.41-6.45, m	7	127.7	6.29, m
*	—	—	8	136.5	6.41-6.45, m	8	137.8	6.40, m
1	195.4	9.43, s	9	142.1	5.97, d (14.7)	9	141.0	6.40, m
2	139.7	—	10	83.7	—	10	135.4	—
3	157.1	6.46, dd (9.6, 1.4)	11	85.1	3.66, d (9.4)	11	138.9	5.61, d (9.5)
4	37.2	2.85-2.94, m	12	43.7	2.00-2.04, m	12	37.8	2.82, qdd (6.8, 9.7, 6.6)
5	81.9	3.86, d (8.0)	13	89.4	3.86, d (9.8)	13	82.6	3.84, d (6.6)
6	133.9	—	14	133.6	—	14	136.3	—
7	135.4	5.24, d (9.6)	15	137.0	5.31, d (9.3)	15	134.1	5.27, d (9.4)
8	31.7	2.38-2.47, m	16	33.0	2.43-2.50, m	16	32.8	2.45, m
9	38.9	1.53-1.70, m	17a	40.5	1.54-1.60, m	17a	40.8	1.55, m
			17b		1.65-1.72, m	17b		1.67, m
10	33.9	2.52-2.62, m	18a	34.8	2.64-2.69, m	18a	34.9	2.64, m
			18b		2.54-2.61, m	18b		2.53, m
11	142.5	—	19	143.9	—	19	144.0	—
12	128.4	7.12-7.19, m	20	129.4	7.19, d (7.2)	20	129.4	7.16, d (6.8)
13	128.3	7.23-7.29, m	21	129.3	7.23-7.28, m	21	129.2	7.24, t (7.5)
14	125.7	7.12-7.19, m	22	126.6	7.15, t (7.2)	22	126.5	7.14, t (7.1)
15	128.3	7.23-7.29, m	23	129.3	7.23-7.28, m	23	129.2	7.24, t (7.5)
16	128.4	7.12-7.19, m	24	129.4	7.19, d (7.2)	24	129.4	7.16, d (6.8)
2-Me	9.6	1.79, d (1.4)	2-Me	13.1	1.99, s	2-Me	13.1	2.01, d (1.0)
4-Me	16.6	0.95, d (6.8)	10-Me	22.3	1.29, s	10-Me	12.9	1.84, s
6-Me	11.5	1.60, d (1.4)	12-Me	13.9	0.99, d (6.5)	12-Me	18.3	0.97, d (6.8)
8-Me	21.0	0.98, d (6.8)	14-Me	11.3	1.63, s	14-Me	12.6	1.61, d (1.0)
*	—	—	16-Me	21.4	0.98, d (6.5)	16-Me	21.5	1.00, d (6.6)
*	—	—	1'-Me	17.1	1.19, d (6.8)	1'-Me	17.1	1.22, d (6.7)
*	—	—	1'	49.8	4.04-4.09, m	1'		4.1, qdd (6.7, 5.5, 11.0)
*	—	—	2'	66.1	3.50-3.58, m	2'	66.1	3.56, ddd (5.5, 11.0, 16.5)

1. W. Trowitzsch-Kienast, E. Forche, V. Wray, H. Reichenbach, E. Jurkiewicz, G. Hunsmann and G. Höfle, *Liebigs Ann. Chem.*, 2006, **1992**, 659-664.

'*' represents that the space left out corresponds to other similar signals of another compound.

^{1}H NMR (500 MHz) and ^{13}C NMR (125 MHz) for compound 1; ^{1}H NMR (600 MHz) and ^{13}C NMR (150 MHz) for compound 3 and phenalamide A2.

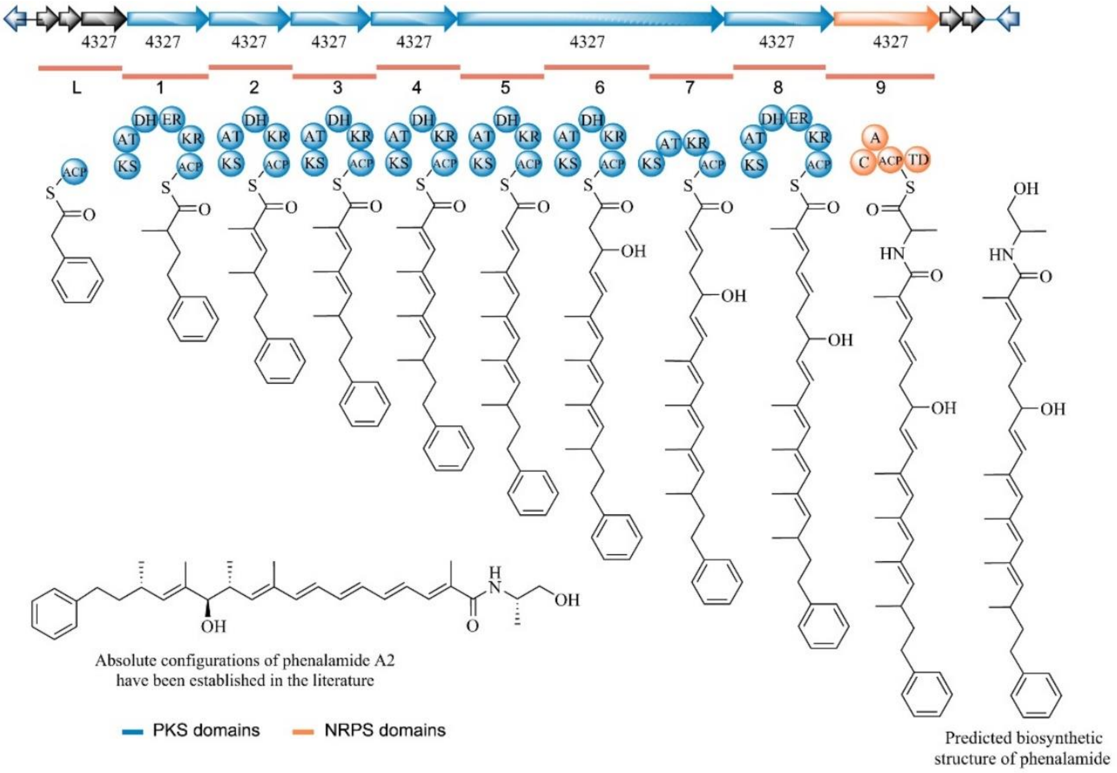

Figure S1 Predicted biosynthetic structure of phenalamide [2].

2. S. Park, H. Hyun, J. S. Lee and K. Cho, *J. Microbiol. Biotechnol.*, 2016, **26**, 1636-1642.

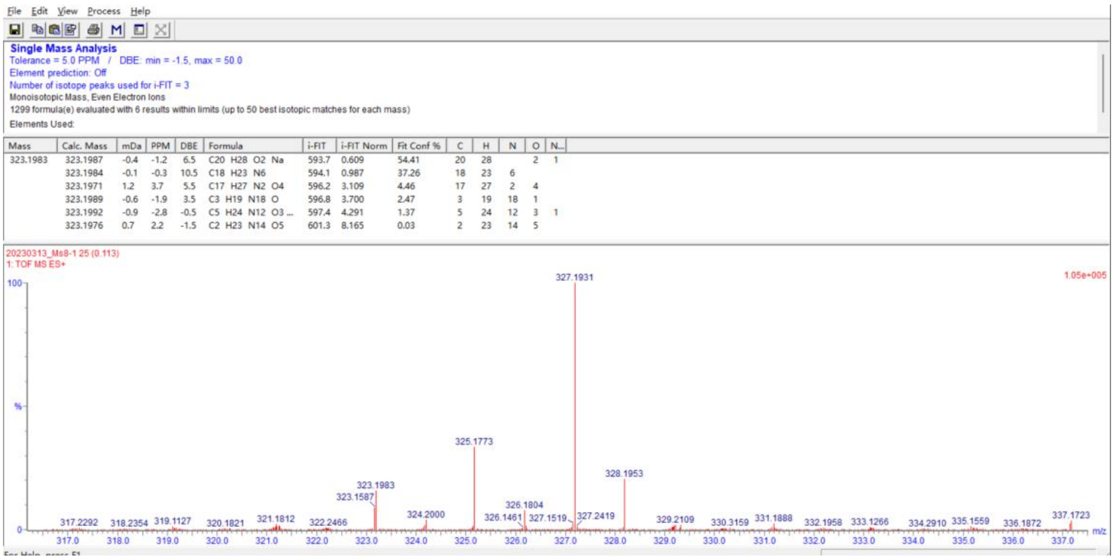

Figure S2 HRESI-MS spectrum of phenalamide D (**1**).

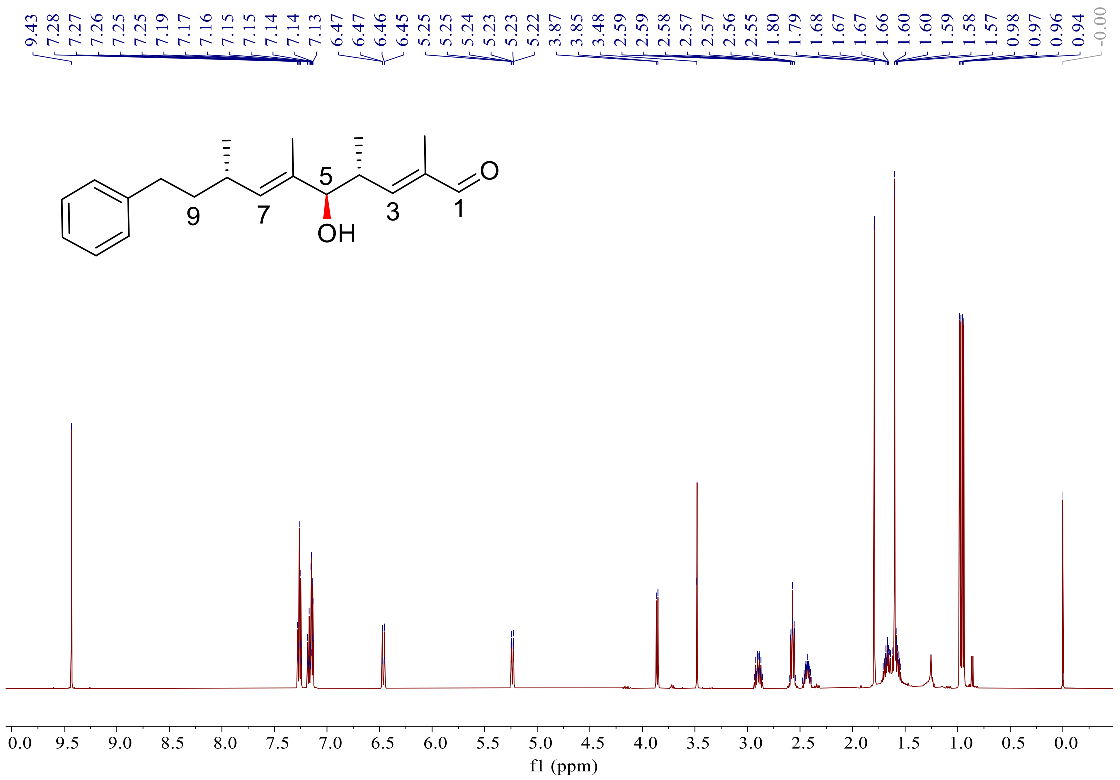

Figure S3 ^{1}H NMR spectrum of phenalamide D (**1**) in $CDCl_3$ (500 MHz).

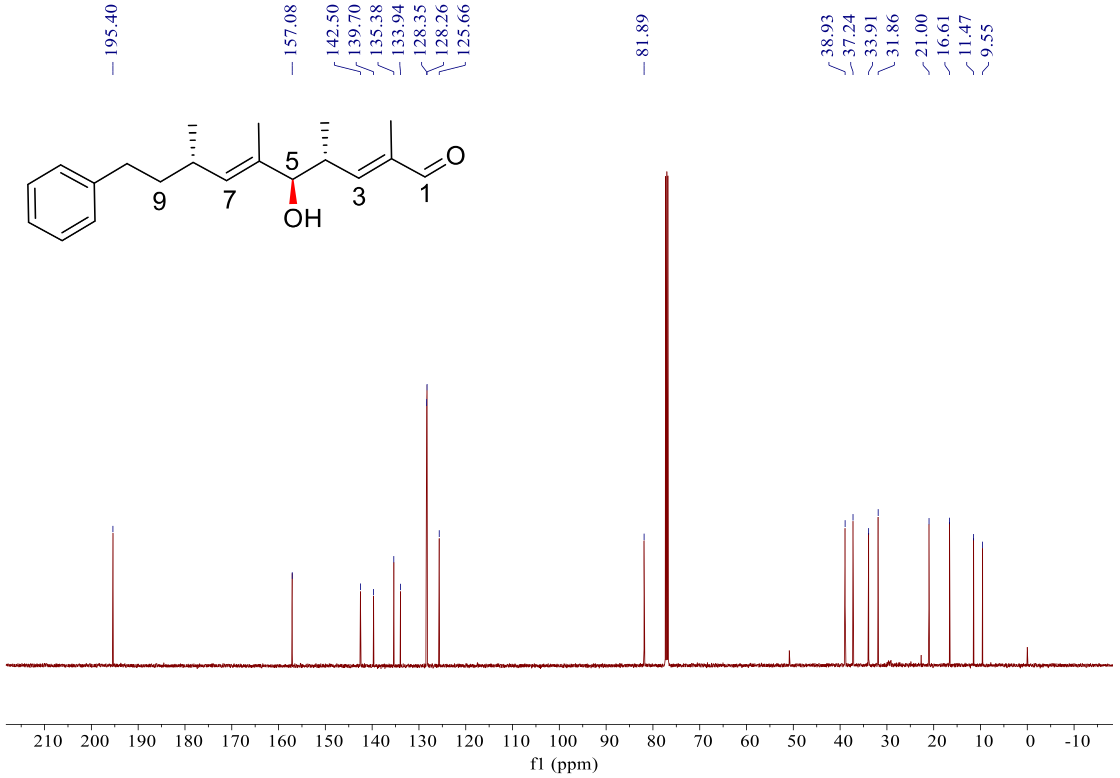

Figure S4 ^{13}C NMR spectrum of phenalamide D (**1**) in $CDCl_3$ (125 MHz).

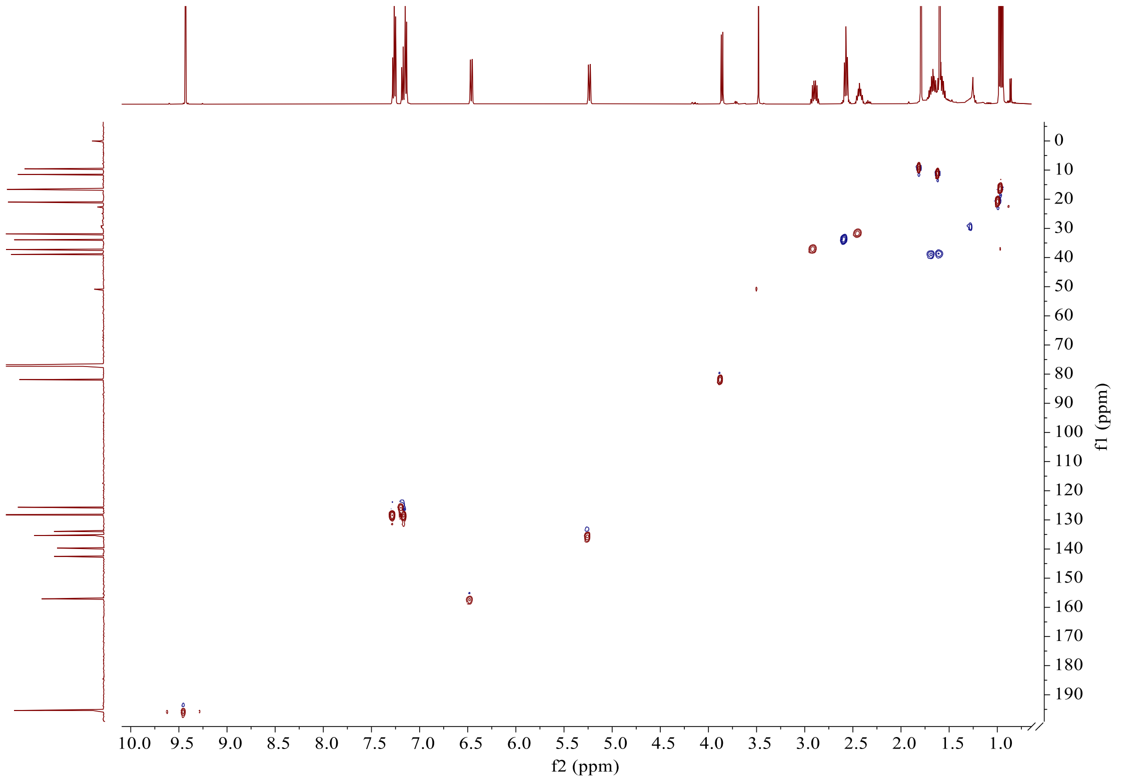

Figure S5 HSQC spectrum of phenalamide D (**1**) in $CDCl_3$.

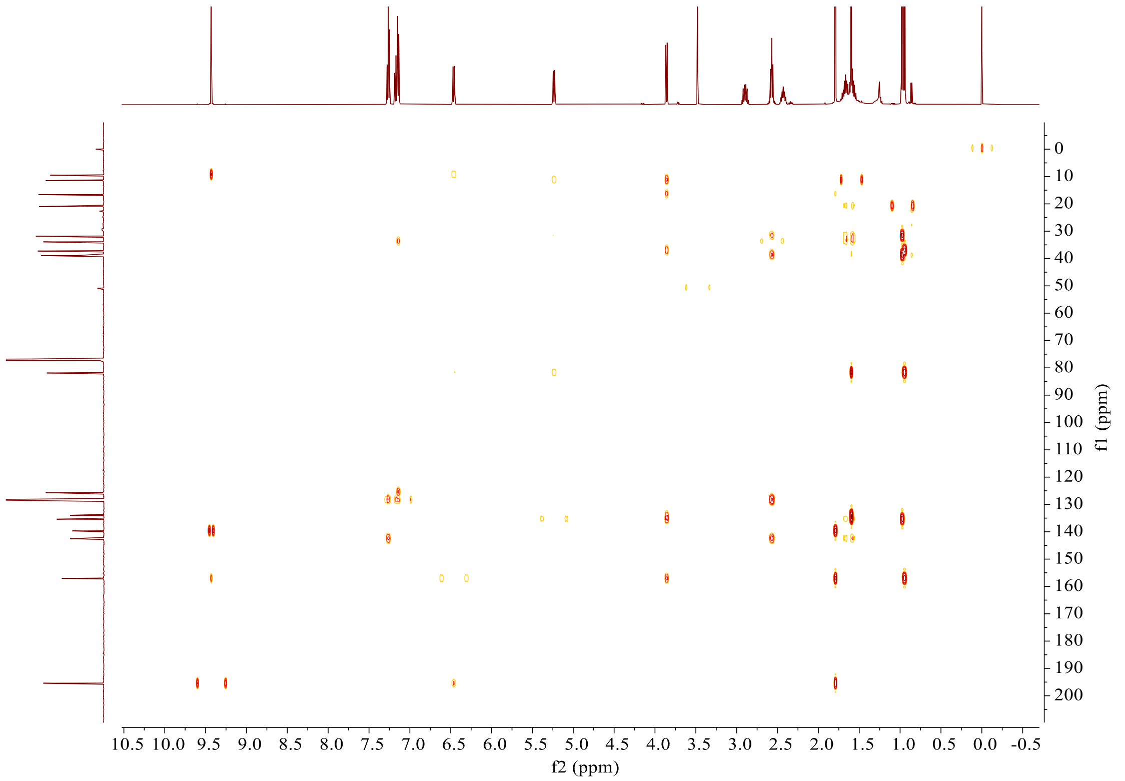

Figure S6 HMBC spectrum of phenalamide D (**1**) in $CDCl_3$.

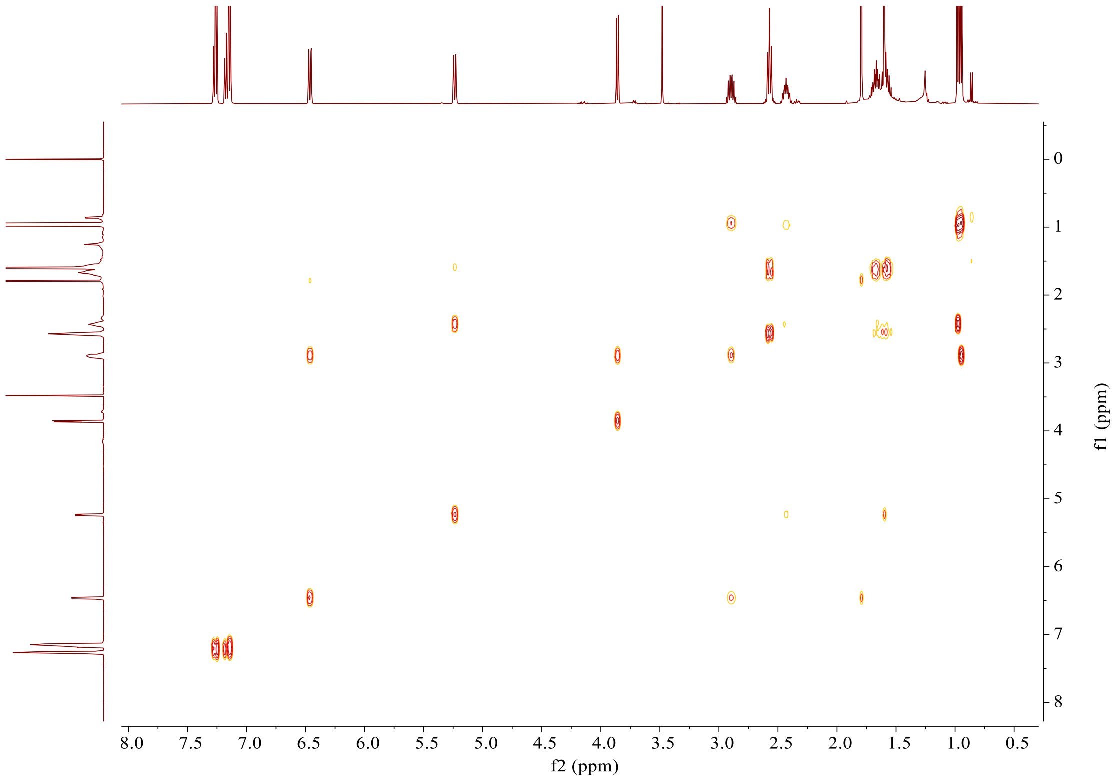

Figure S7 ^{1}H-^{1}H COSY spectrum of phenalamide D (**1**) in $CDCl_3$.

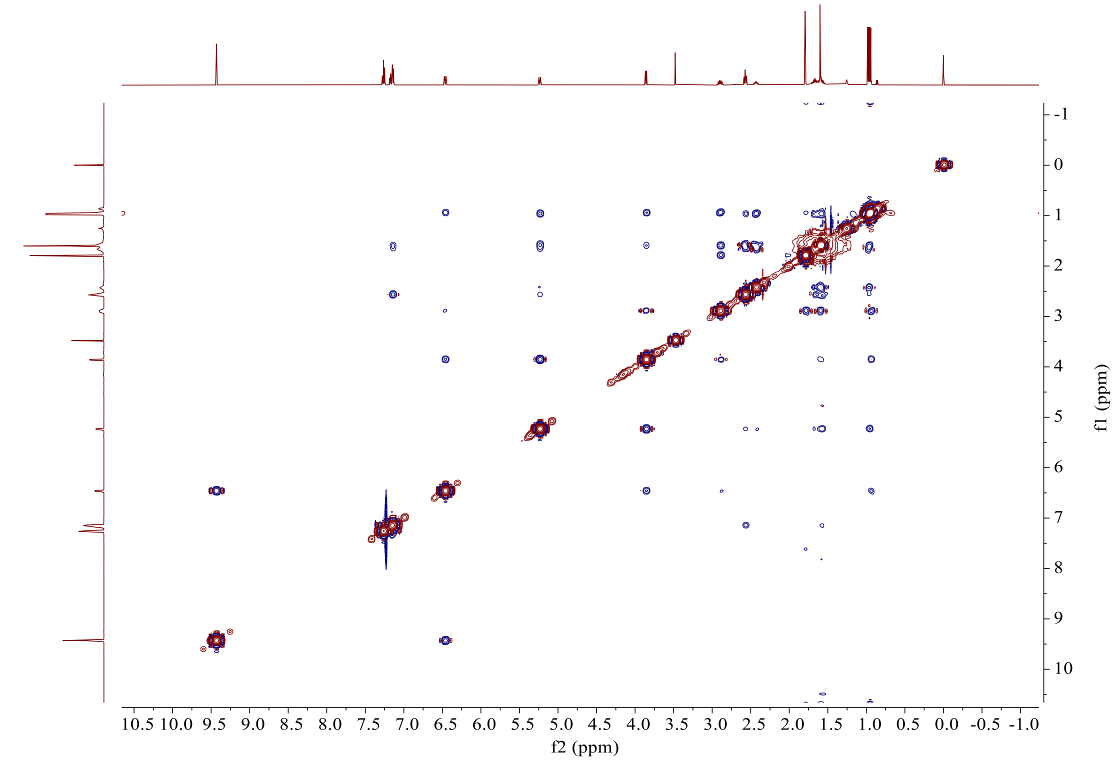

Figure S8 NOESY spectrum of phenalamide D (**1**) in $CDCl_3$.

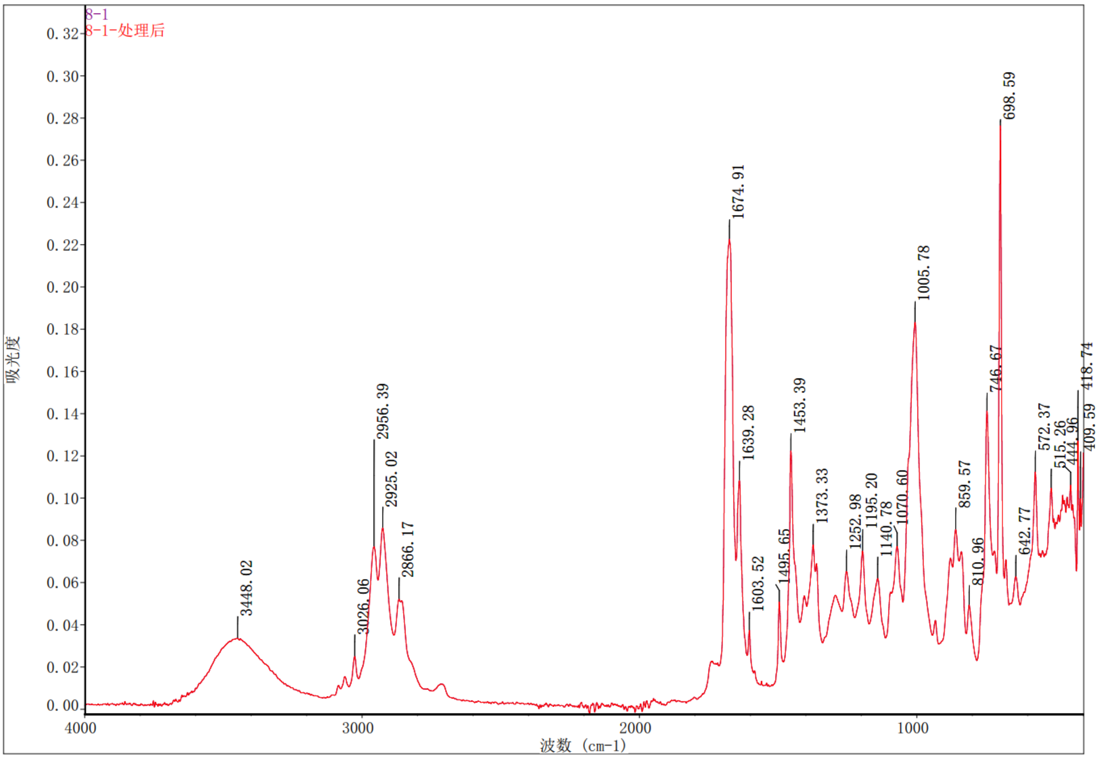

Figure S9 IR spectrum of phenalamide D (**1**).

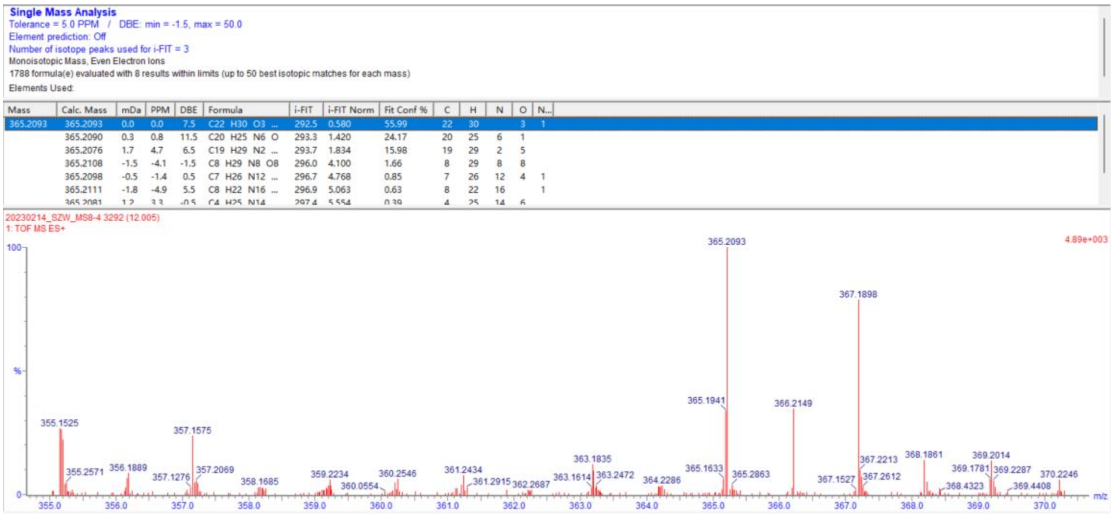

Figure S10 HRESI-MS spectrum of phenalamide E (**2**).

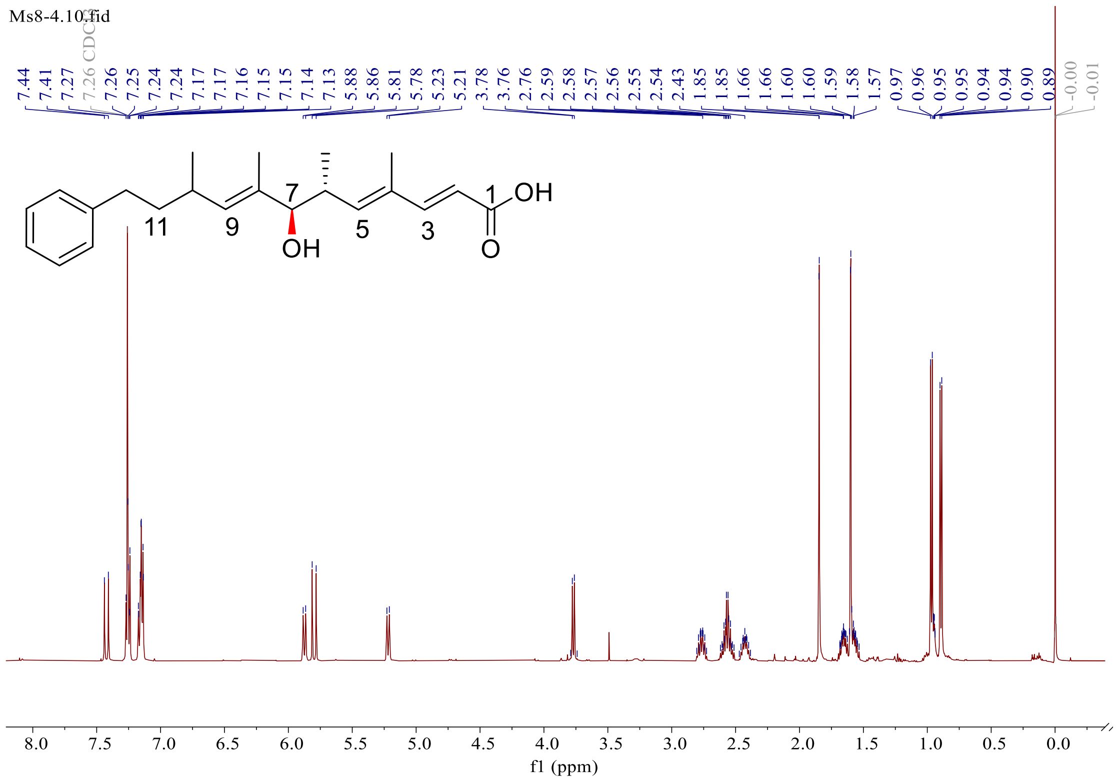

Figure S11 ^{1}H NMR spectrum of phenalamide E (**2**) in $CDCl_3$ (500 MHz).

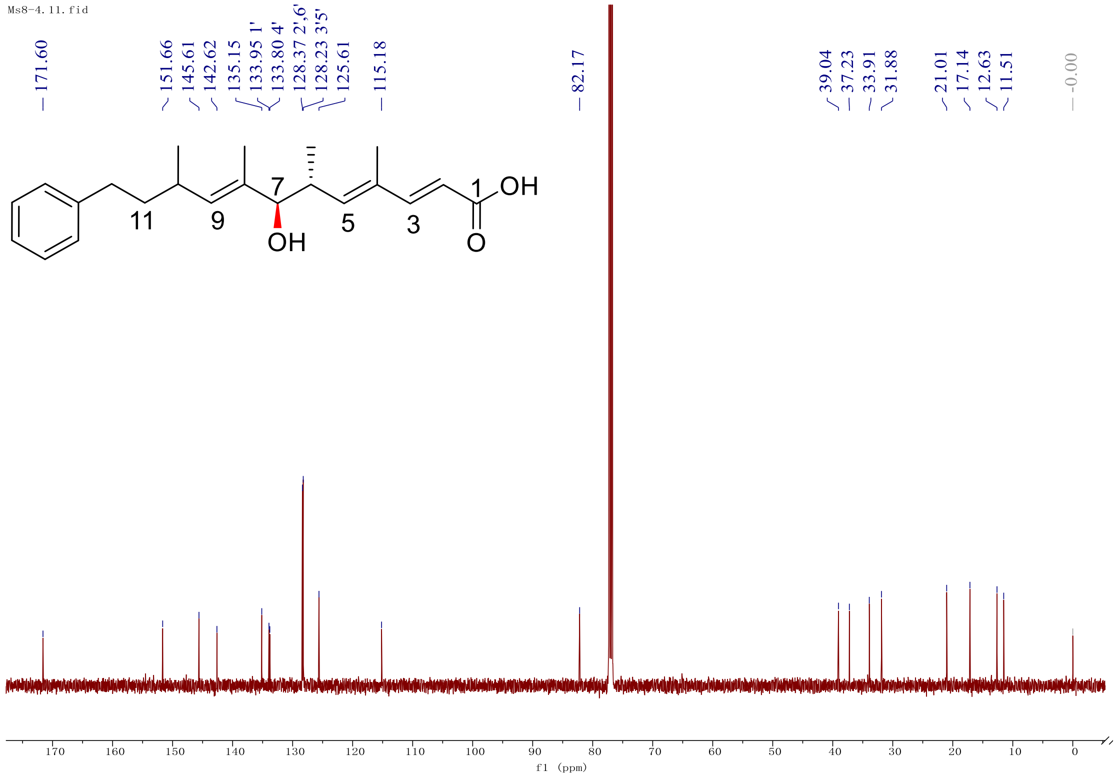

Figure S12 ^{13}C NMR spectrum of phenalamide E (**2**) in $CDCl_3$ (125 MHz).

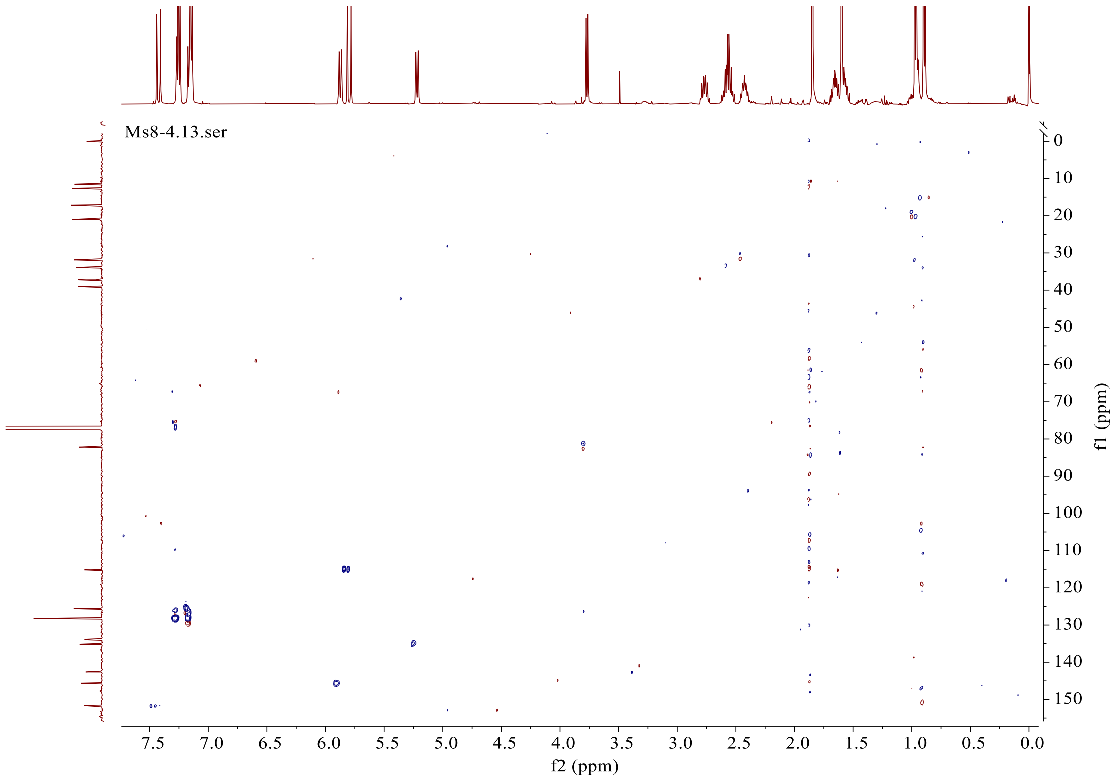

Figure S13 HSQC spectrum of phenalamide E (**2**) in $CDCl_3$.

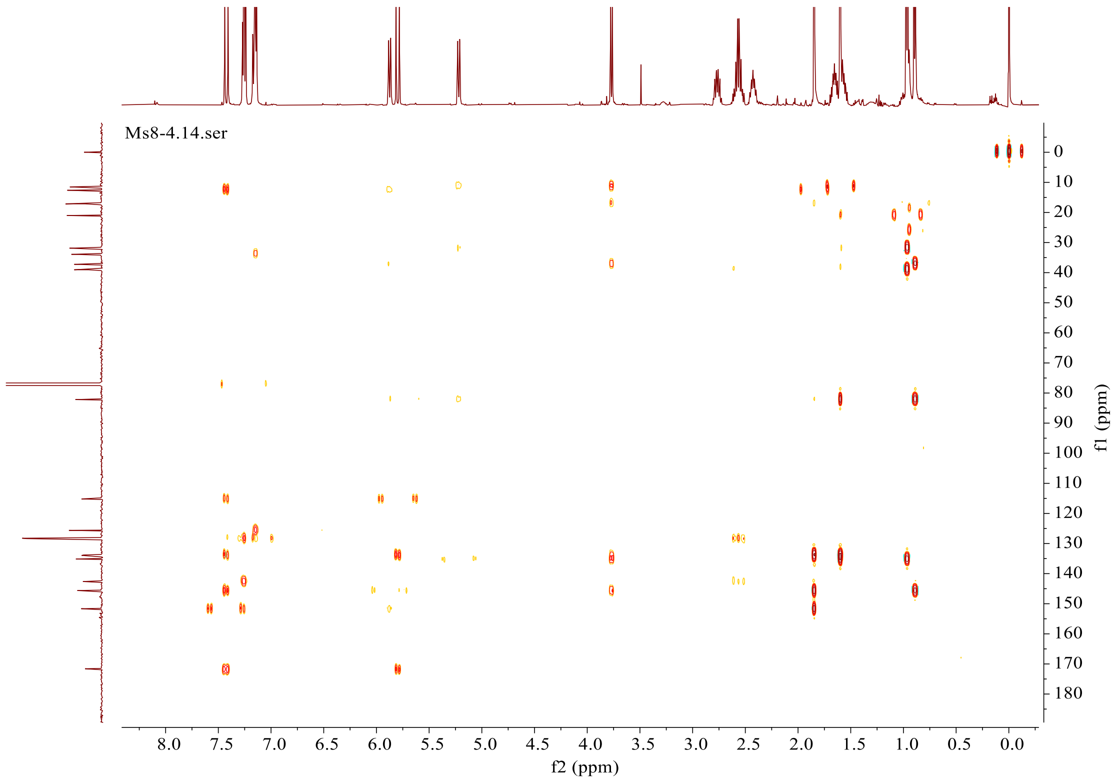

Figure S14 HMBC spectrum of phenalamide E (**2**) in $CDCl_3$.

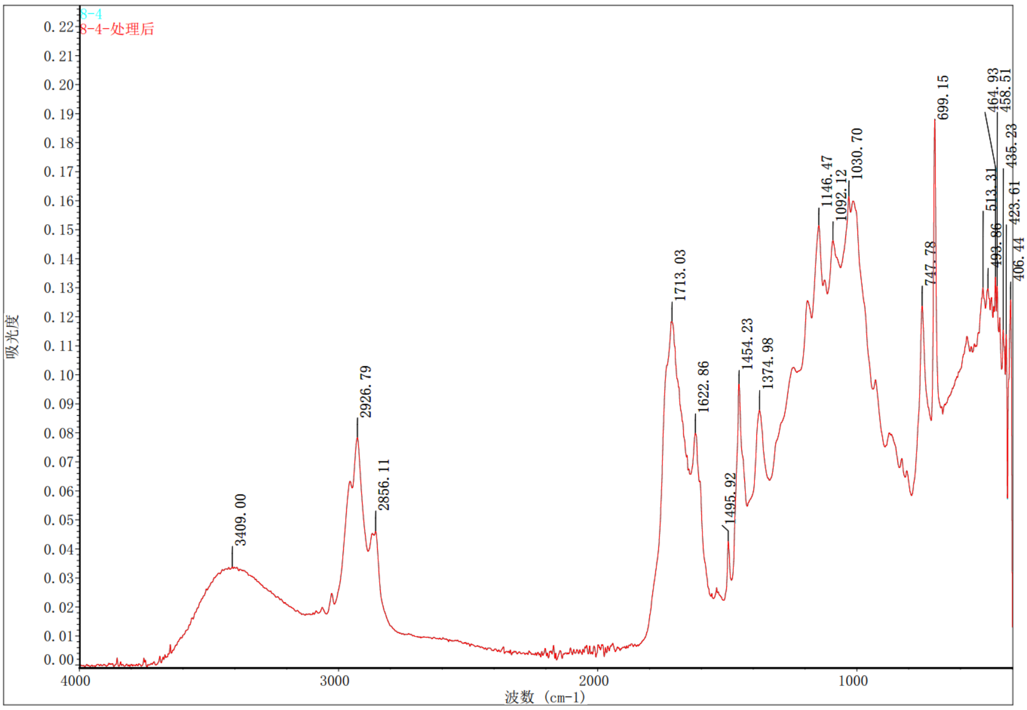

Figure S15 IR spectrum of phenalamide E (**2**).

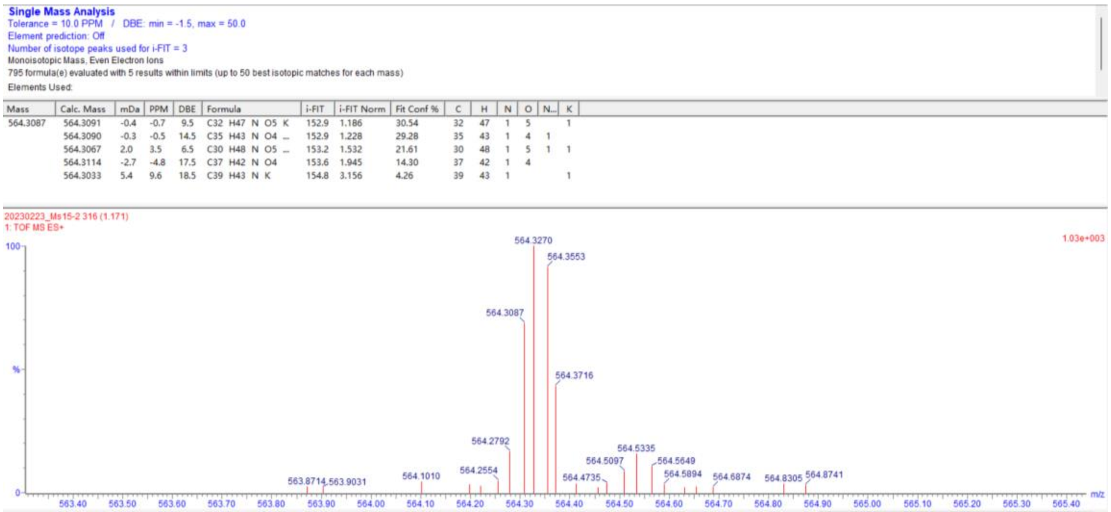

Mass	Calc. Mass	mDa	PPM	DBE	Formula	i-FIT	i-FIT Norm	Fit Conf %	C	H	N	O	N...	K
564.3087	564.3091	-0.4	-0.7	9.5	C32 H47 N O5 K	152.9	1.186	30.54	32	47	1	5		1
	564.3090	-0.3	-0.5	14.5	C35 H43 N O4 ...	152.9	1.228	29.28	35	43	1	4	1	
	564.3067	2.0	3.5	6.5	C30 H48 N O5 ...	153.2	1.532	21.61	30	48	1	5	1	1
	564.3114	-2.7	-4.8	17.5	C37 H42 N O4	153.6	1.945	14.30	37	42	1	4		
	564.3033	5.4	9.6	18.5	C39 H43 N K	154.8	3.156	4.26	39	43	1			1

Figure S16 HRESI-MS spectrum of phenalamide F (**3**).

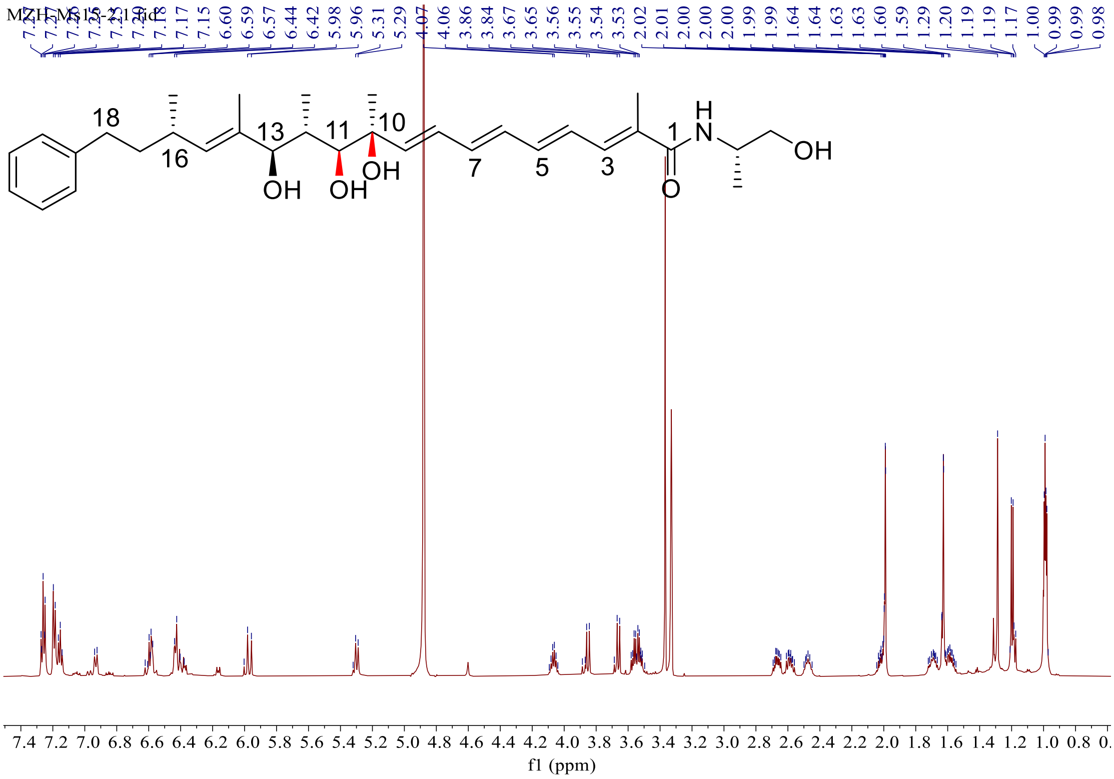

Figure S17 ^{1}H NMR spectrum of phenalamide F (**3**) in CD_3OD (600 MHz).

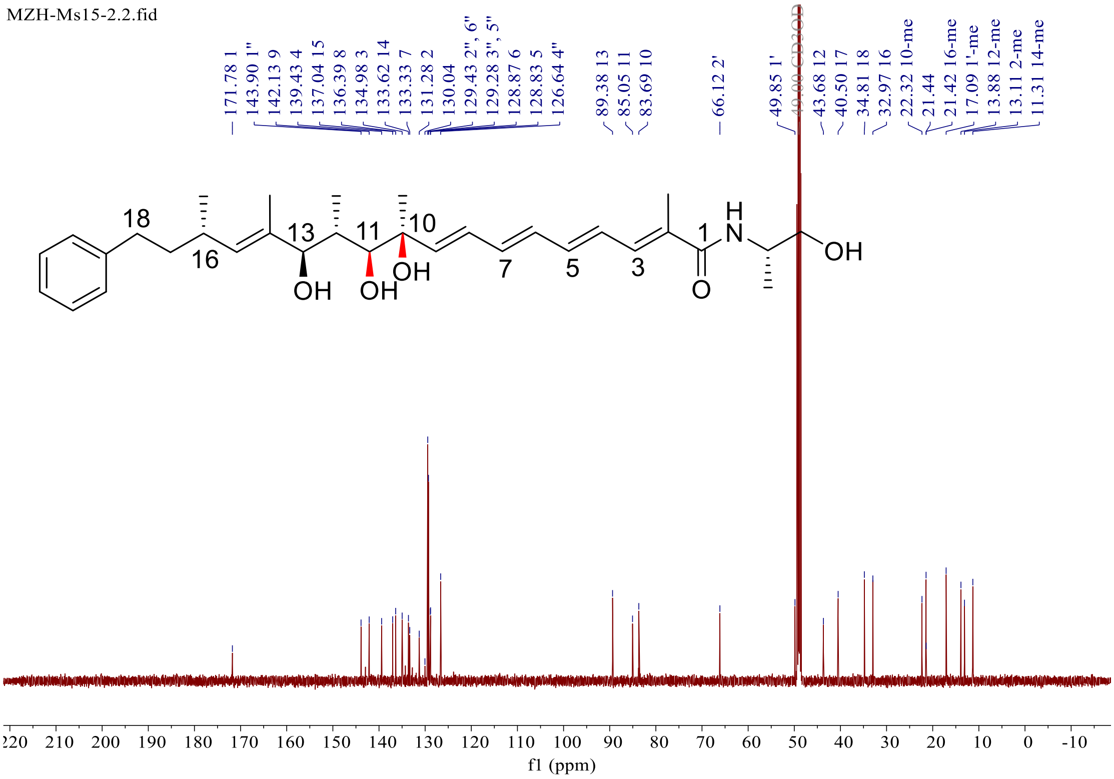

Figure S18 ^{13}C NMR spectrum of phenalamide F (**3**) in CD_3OD (150 MHz).

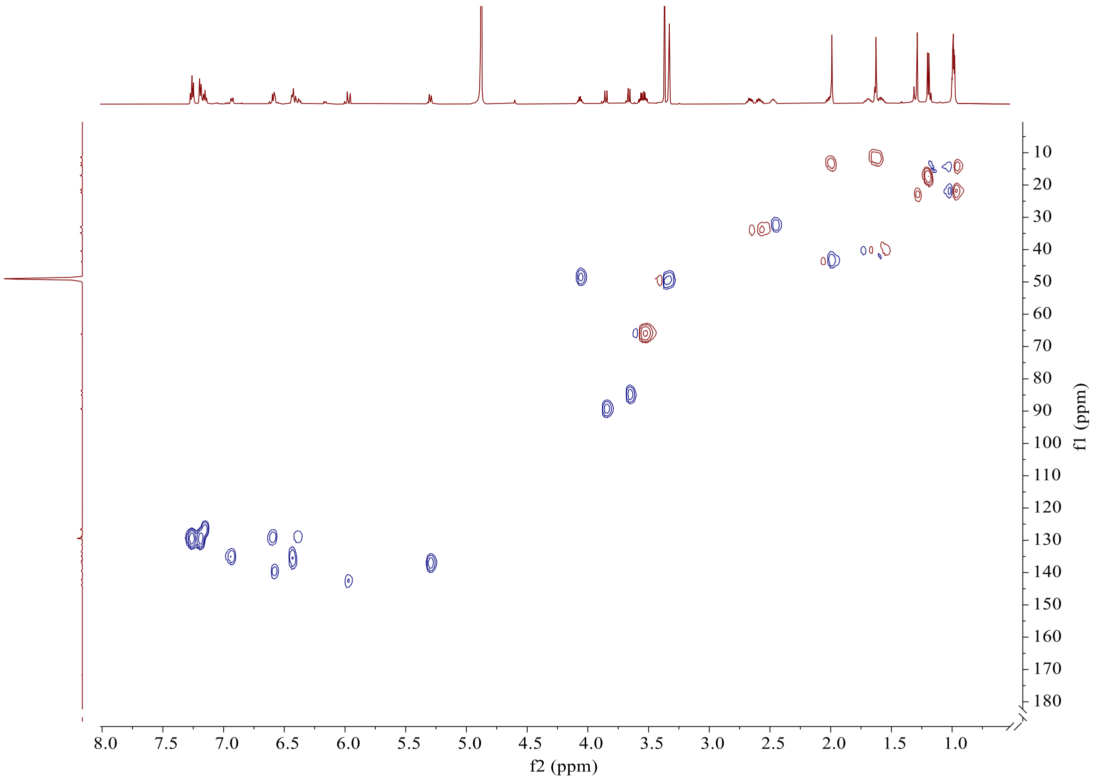

Figure S19 HSQC spectrum of phenalamide F (**3**) in CD_3OD.

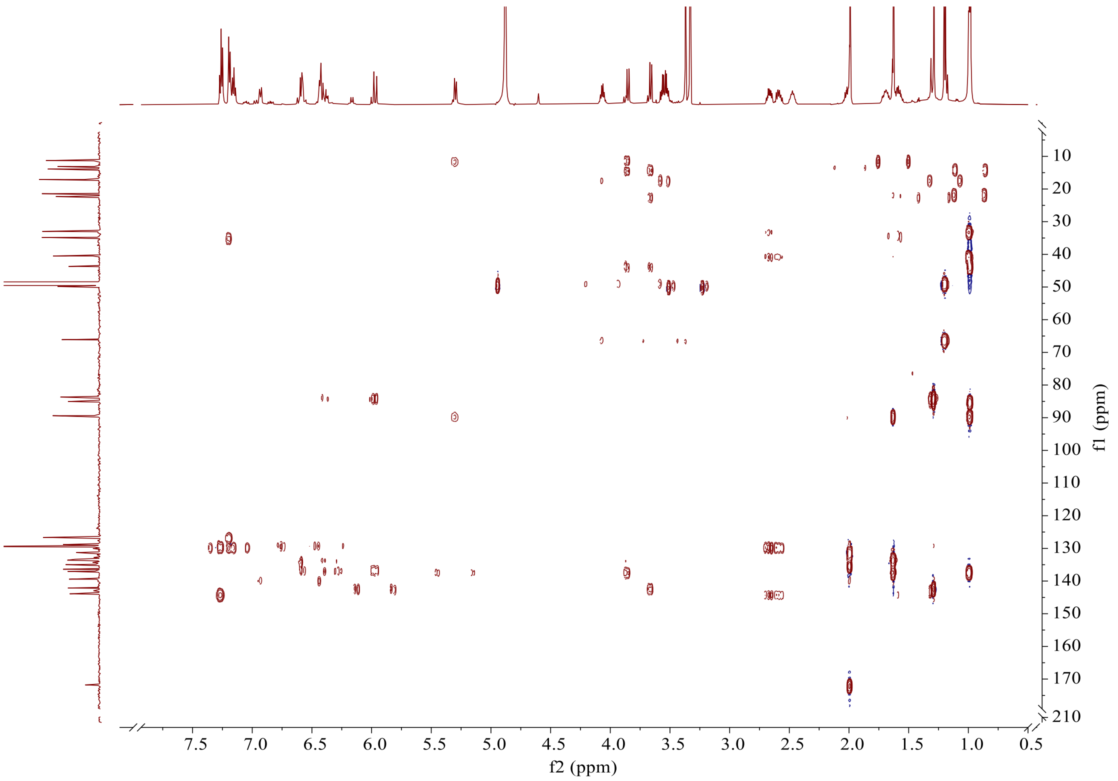

Figure S20 HMBC spectrum of phenalamide F (**3**) in CD_3OD.

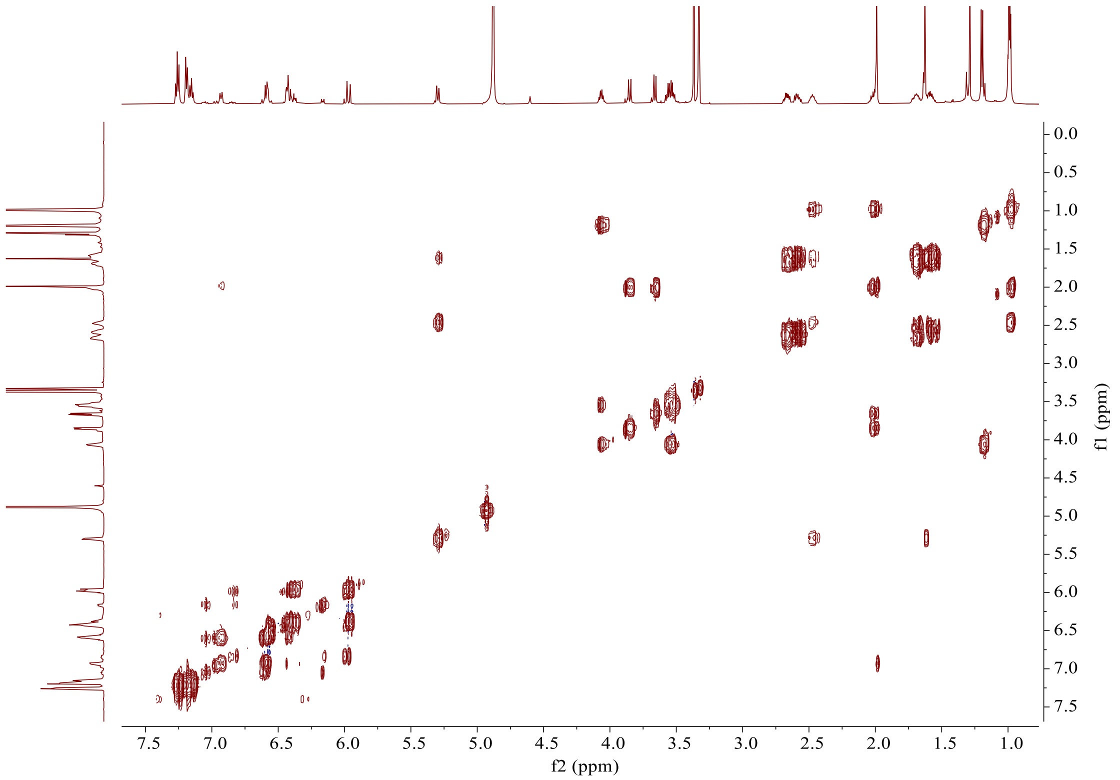

Figure S21 ^{1}H-^{1}H COSY spectrum of phenalamide F (**3**) in CD_3OD.

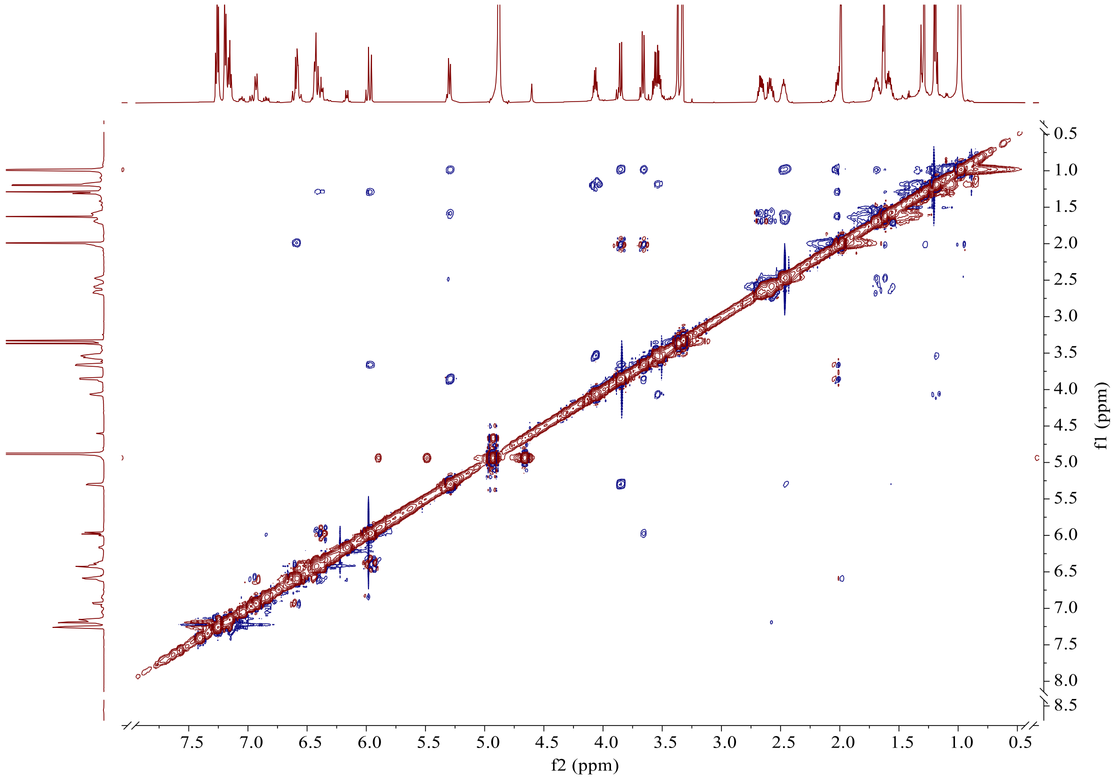

Figure S22 NOESY spectrum of phenalamide F (**3**) in CD_3OD.

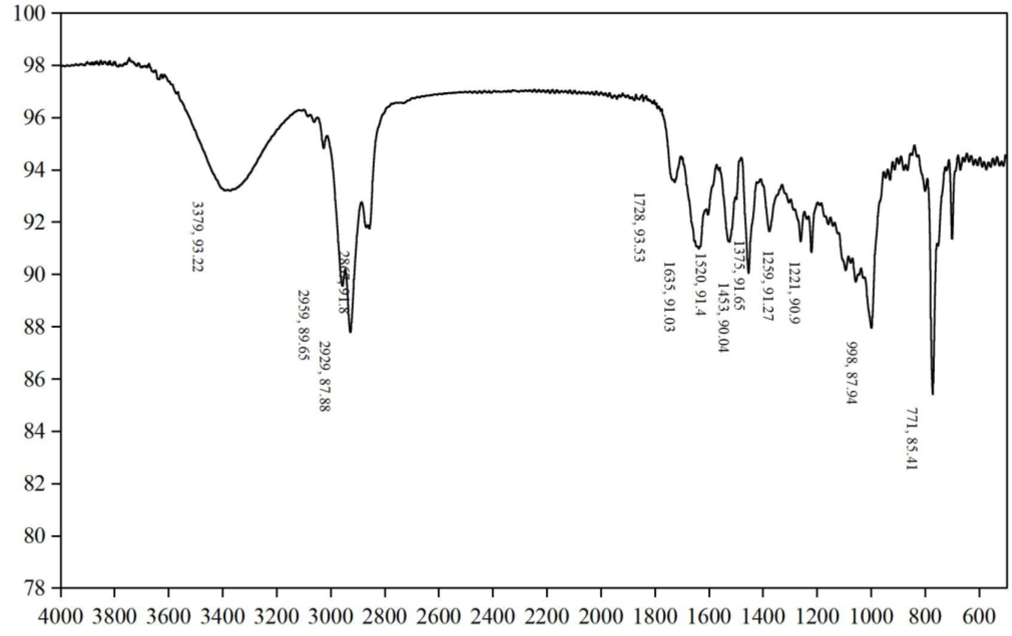

Figure S23 IR spectrum of phenalamide F (**3**).

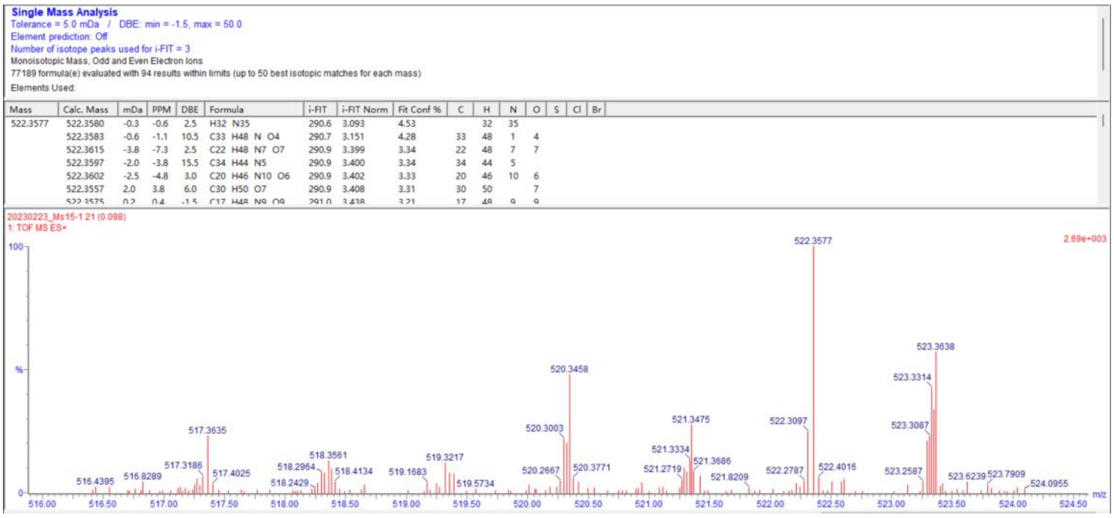

Figure S24 HRESI-MS spectrum of phenalamide G (**4**).

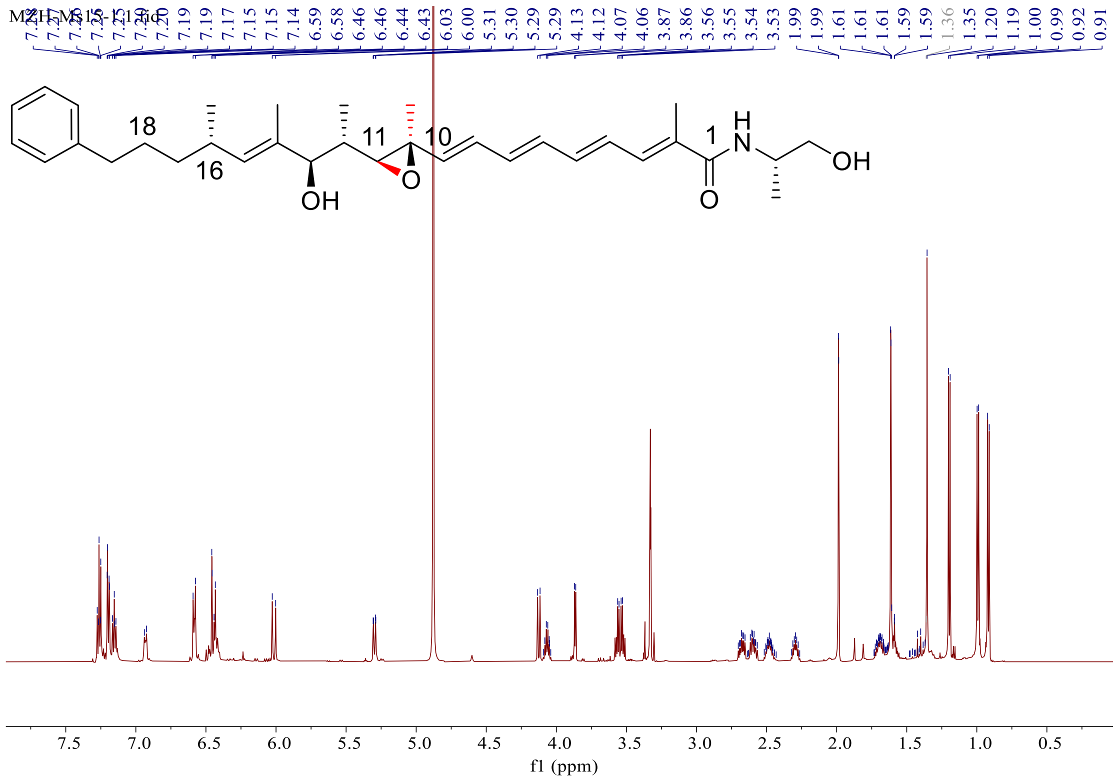

Figure S25 ^{1}H NMR spectrum of phenalamide G (**4**) in CD_3OD (600 MHz).

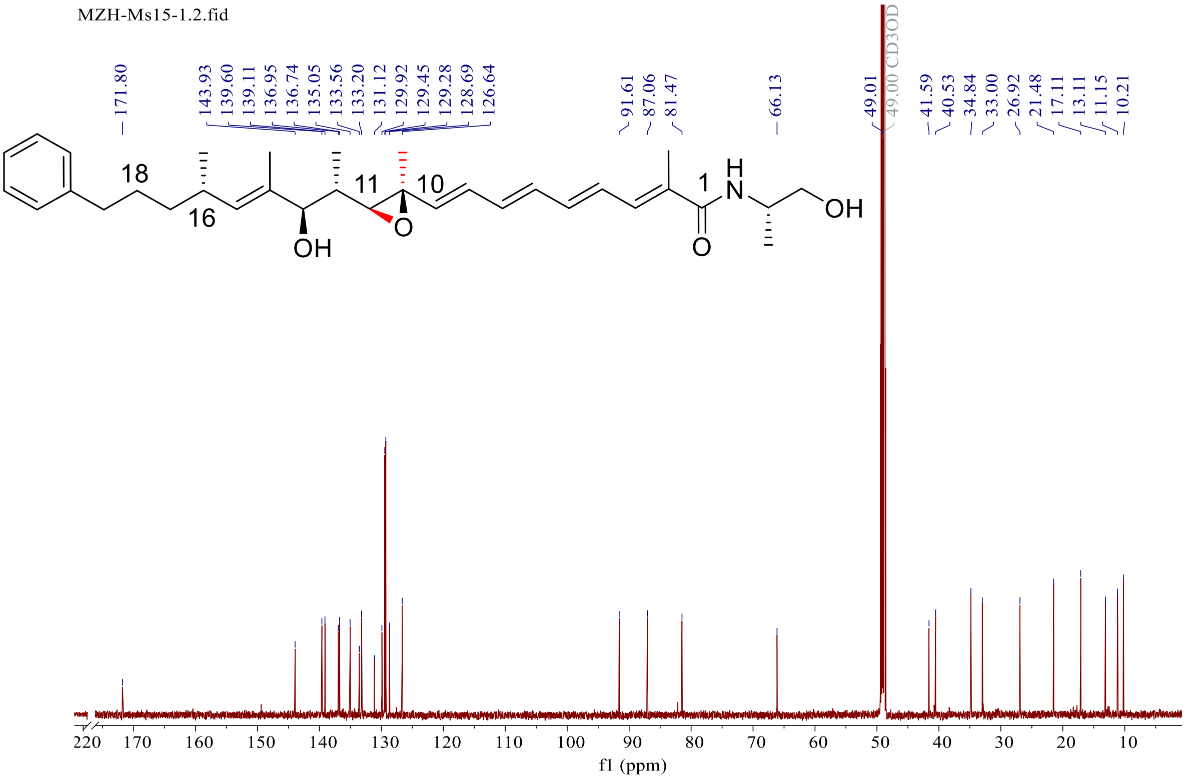

Figure S26 ^{13}C NMR spectrum of phenalamide G (**4**) in CD_3OD (150 MHz).

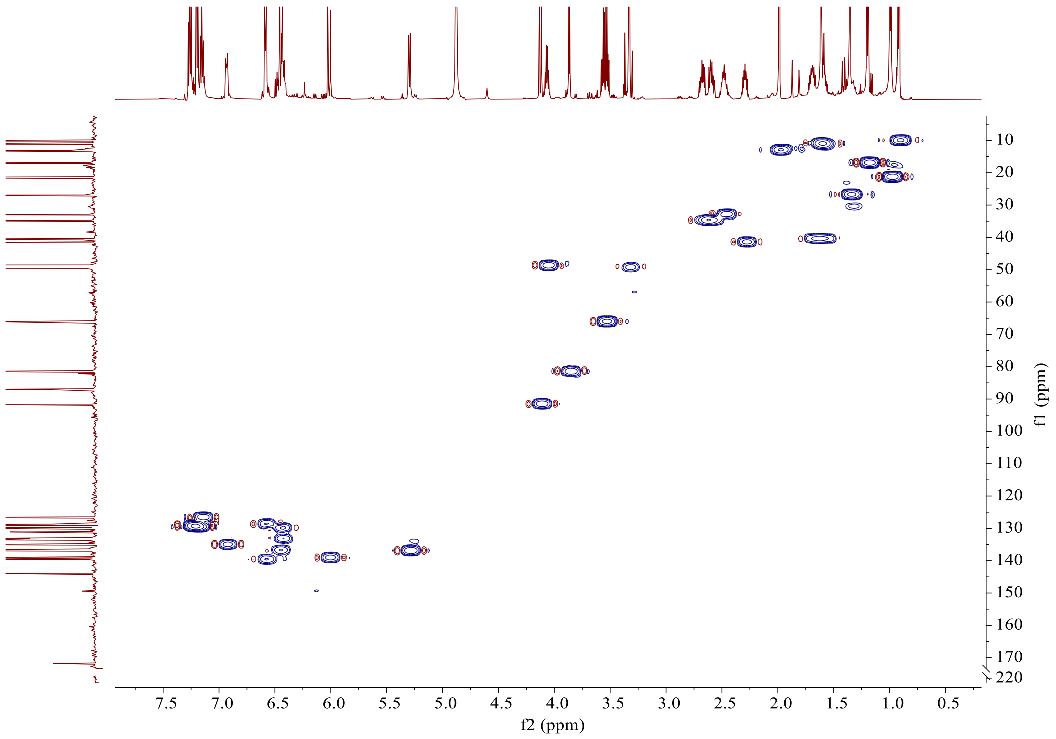

Figure S27 HSQC spectrum of phenalamide G (**4**) in CD_3OD.

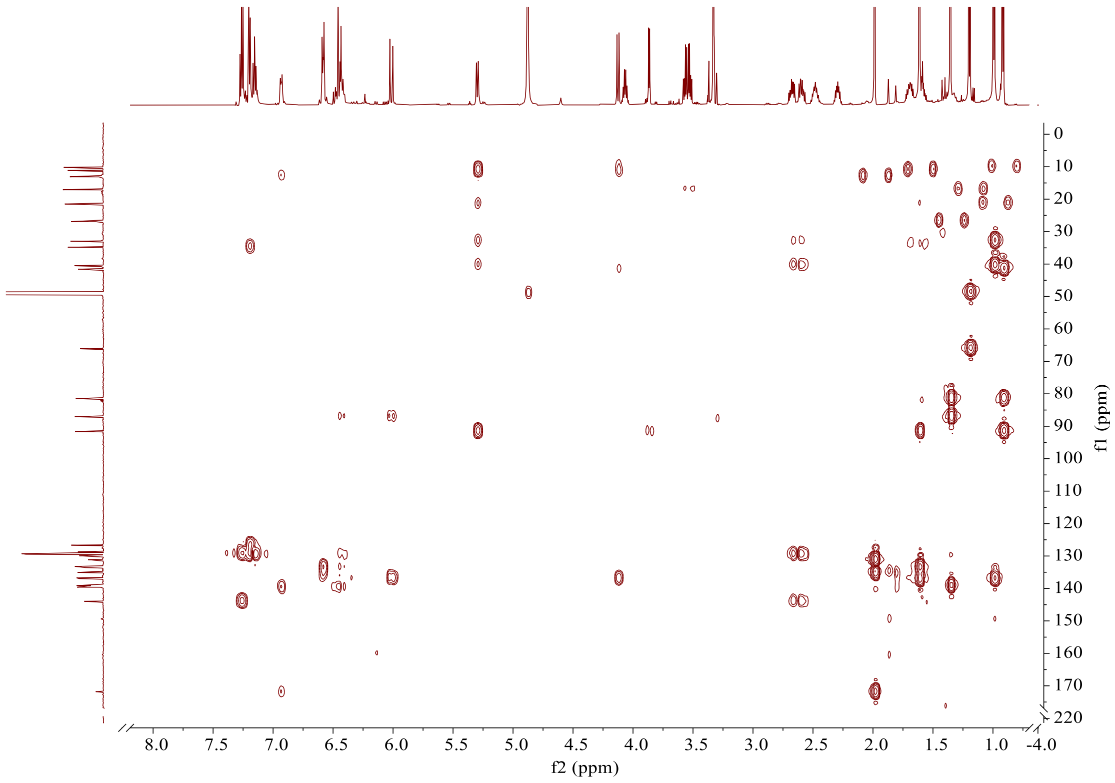

Figure S28 HMBC spectrum of phenalamide G (**4**) in CD_3OD.

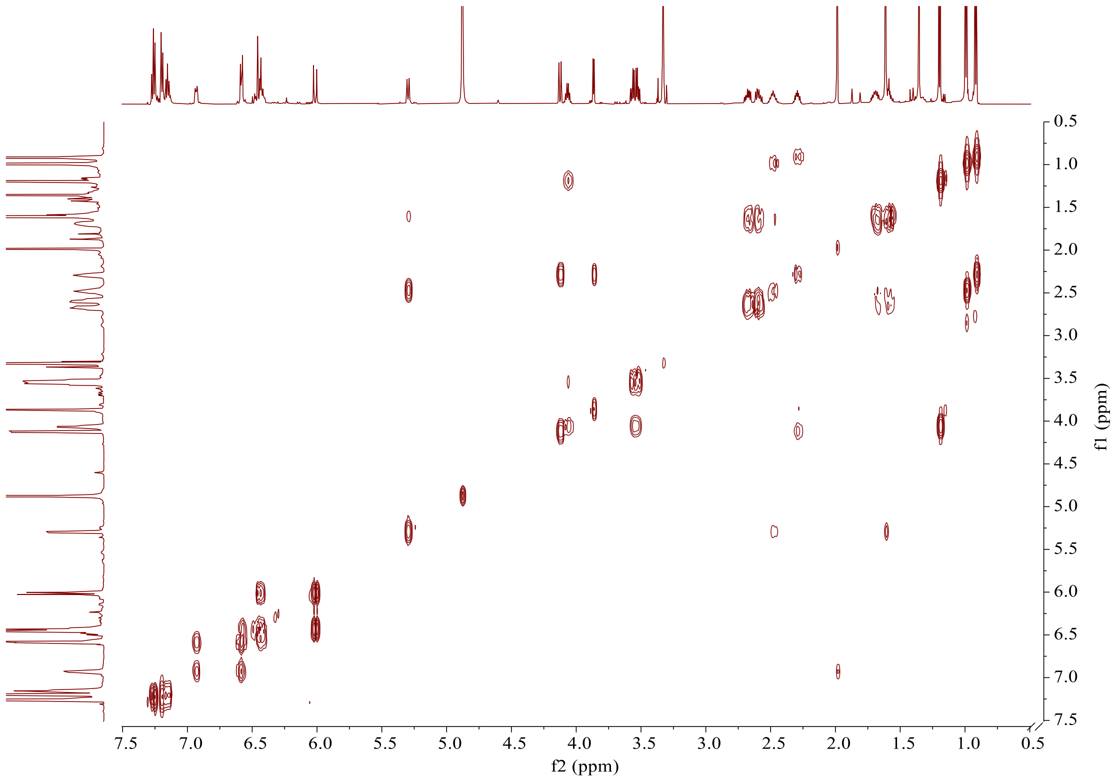

Figure S29 ^{1}H-^{1}H COSY spectrum of phenalamide G (**4**) in CD_3OD.

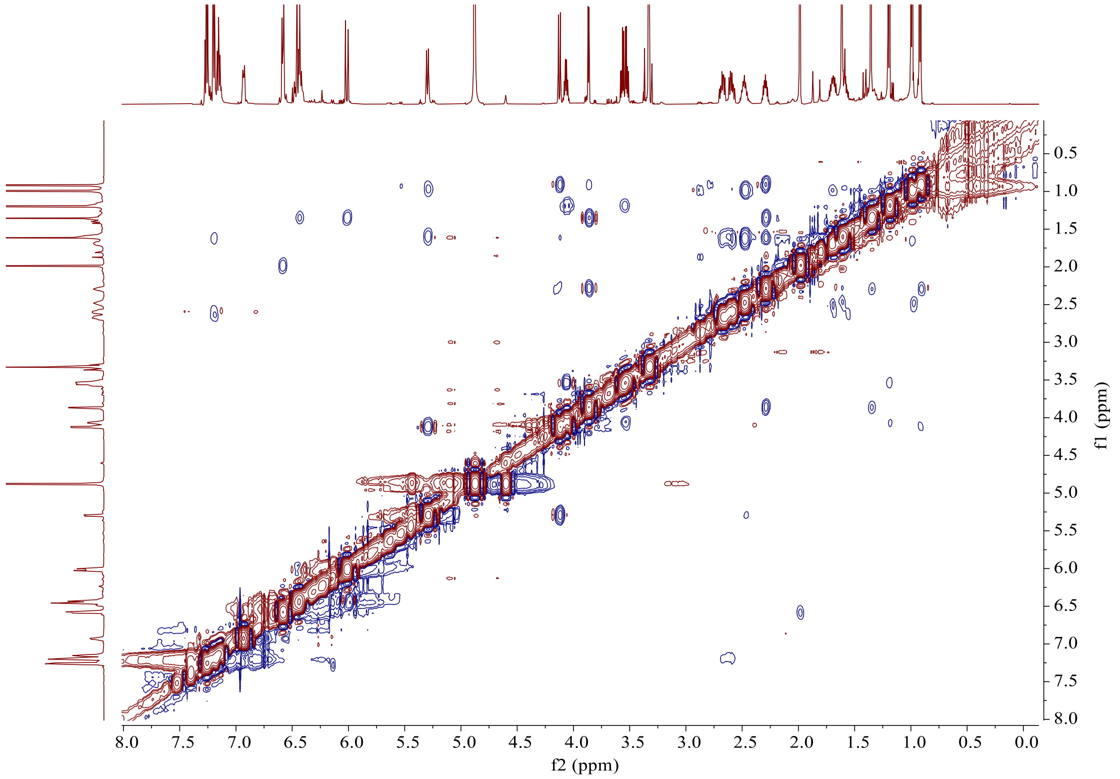

Figure S30 NOESY spectrum of phenalamide G (**4**) in CD_3OD.

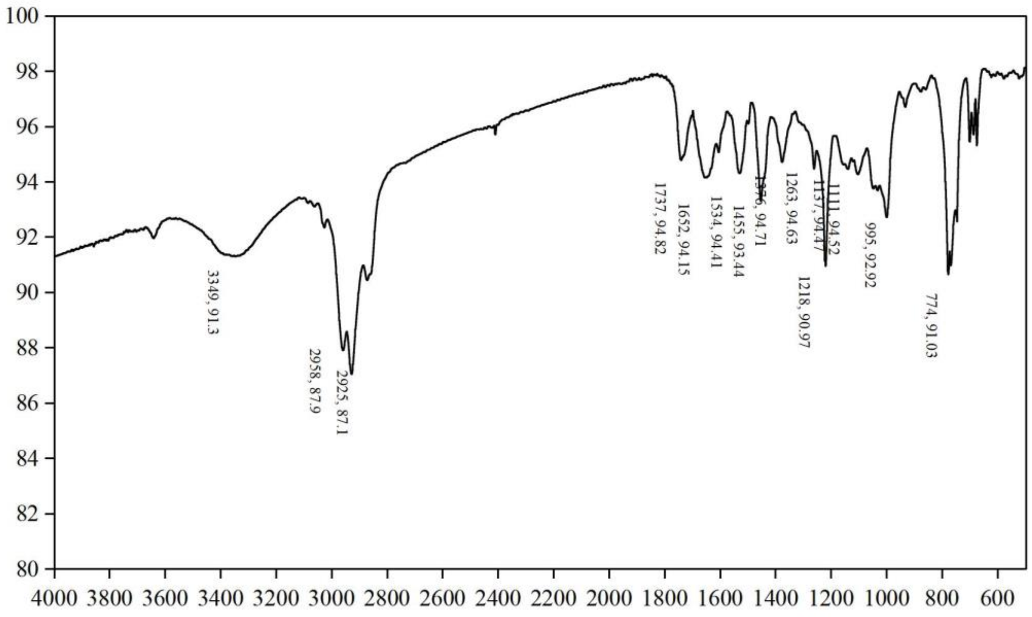

Figure S31 IR spectrum of phenalamide G (**4**).

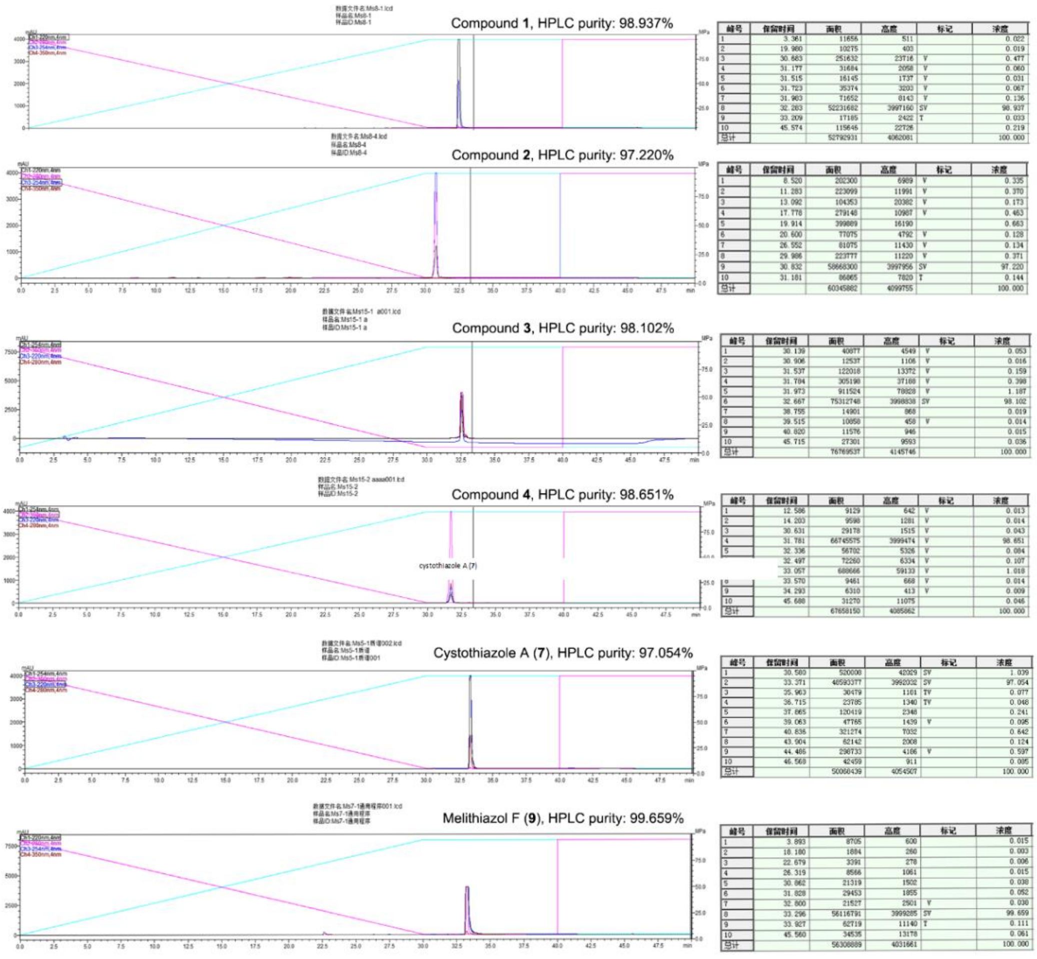

Figure S32. HPLC purity chromatograms of the isolated new compounds and potent active.
